# Nationwide organ volume distributions and cross-sectional age-associated differences in abdominal CT from Japan

**DOI:** 10.1007/s11604-026-02028-z

**Published:** 2026-06-11

**Authors:** Tomohiro Kikuchi, Kohei Yamamoto, Yosuke Yamagishi, Toshiaki Akashi, Shouhei Hanaoka, Takeharu Yoshikawa, Hiroyuki Fujii, Harushi Mori, Hisaki Makimoto, Takahide Kohro

**Affiliations:** 1 Data Science Center, Jichi Medical University, 3311-1 Yakushiji, Shimotsuke, Tochigi, 329-0498 Japan; 2 Department of Radiology, Jichi Medical University, 3311-1 Yakushiji, Shimotsuke, Tochigi, 329-0498 Japan; 3 Department of Computational Diagnostic Radiology and Preventive Medicine, The University of Tokyo Hospital, 7-3-1 Hongo, Bunkyo-Ku, Tokyo, 113-8655 Japan; 4 Division of Radiology and Biomedical Engineering, Graduate School of Medicine, The University of Tokyo, 7-3-1 Hongo, Bunkyo-Ku, Tokyo, 113-8655 Japan; 5 Department of Radiology, Juntendo University School of Medicine, 2-1-1 Hongo, Bunkyo-Ku, Tokyo, 113-8421 Japan; 6 Department of Radiology, The University of Tokyo Hospital, 7-3-1 Hongo, Bunkyo-Ku, Tokyo, 113-8655 Japan

**Keywords:** Computed tomography, Abdomen, Reference values, Organ volume, Segmentation

## Abstract

**Purpose:**

Large-scale CT-based reference distributions for abdominal organ volume that incorporate age, sex, and body size are limited. To establish sex- and age-specific reference distributions for major abdominal organ volumes on non-contrast abdominopelvic CT in a nationwide Japanese cohort to provide a foundation for automated clinical assessment.

**Materials and methods:**

In this retrospective, multicenter study, we used the Japan Medical Image Database to identify all non-contrast abdominopelvic CT examinations performed in 2024. Unique adults with available data on age, sex, height, and weight were included in this study. The final sample comprised 49,764 examinations (26,456 men and 23,308 women) conducted at nine different institutions. Automated segmentation (TotalSegmentator v2.10.0) was used to produce organ volumes, excluding the hollow viscera. Sex-specific 10th, 25th, 50th, 75th, and 90th percentiles were calculated. Age–volume relationships of body surface area (BSA)-normalized volumes (mL/m^2^) were modeled using natural cubic splines (four degrees of freedom) separately for each sex.

**Results:**

The median male vs. female volumes (mL) were as follows: liver, 1,194.7 vs 1,024.0; pancreas, 63.7 vs 52.3; spleen, 118.7 vs 95.4; kidneys (total), 268.3 vs 221.2; adrenals (total), 6.7 vs 4.2; iliopsoas (total), 483.4 vs 317.7; and prostate, 25.5 (men only). The age–volume relationships of BSA-normalized volumes showed convex patterns for the liver, pancreas, and kidneys in both sexes and for male adrenal glands; lower values in older age groups for the spleen and iliopsoas in both sexes; and higher values in older age groups for the prostate and female adrenal glands.

**Conclusion:**

This nationwide Japanese CT cohort provides sex- and age-resolved organ volume reference distributions. These distributions may serve as a quantitative reference for clinical interpretation and population-based imaging research.

**Supplementary Information:**

The online version contains supplementary material available at https://doi.org/10.1007/s11604-026-02028-z.

## Introduction

Advances in computer vision and data science have expanded medical image analysis beyond subjective visual reads to standardized quantitative outputs, including organ volumes, CT attenuation, radiomic descriptors, and AI-derived metrics, yielding clinically useful insights [1, 2]. In particular, organ volumetry is linked to functional assessment, disease course, and prognosis. For example, total kidney volume predicts functional prognosis in autosomal dominant polycystic kidney disease, preoperative liver volume predicts post-hepatectomy outcomes, and pancreatic volume changes are associated with diabetes, underscoring its value as an imaging biomarker [3–6]. Reference distributions describing organ volume distributions in large cohorts are therefore essential as a foundational infrastructure for quantitative imaging. Previous CT-based studies, typically involving several hundred to a few thousand individuals, have reported reference values for individual abdominal organs [7–11]. Although recent pioneering studies have successfully utilized automated segmentation in relatively large cohorts [10, 11], many studies in this field have historically relied on manual or semi-automated methods [7–9], which often limit reproducibility and cross-study comparability. Furthermore, nationwide reference data covering multiple abdominal organs and constructed using a single, standardized, and fully automated pipeline remain scarce [12].

The Japan Medical Image Database (J-MID) is a national cloud-based platform that continuously aggregates routine CT and MR images from 11 major university hospitals in Japan [13]. Leveraging this resource, we extracted non-contrast abdominopelvic CT examinations and applied TotalSegmentator, a publicly available and fully automated deep learning-based segmentation framework, to compute volumes for multiple abdominal organs using a single, consistent pipeline [14].

Using tens of thousands of examinations, we derived sex- and age-specific organ volume distributions that can serve as a quantitative reference for clinical interpretation, epidemiological studies, and downstream AI applications, including automated reporting.

## Methods

### Study design and data source

This study was performed in line with the principles of the Declaration of Helsinki. This study was approved by the Institutional Review Board of the participating institution (approval number: J24-017). The requirement for written informed consent was waived because of the retrospective nature of this study. This study was conducted following the STROBE (Strengthening the Reporting of Observational Studies in Epidemiology) guidelines [18].

In this retrospective multicenter study, we used data from the J-MID, a nationwide repository that aggregates CT and MRI examinations, together with radiology reports from 11 university hospitals across Japan [13].

### Patient selection

**Fig. 1:**
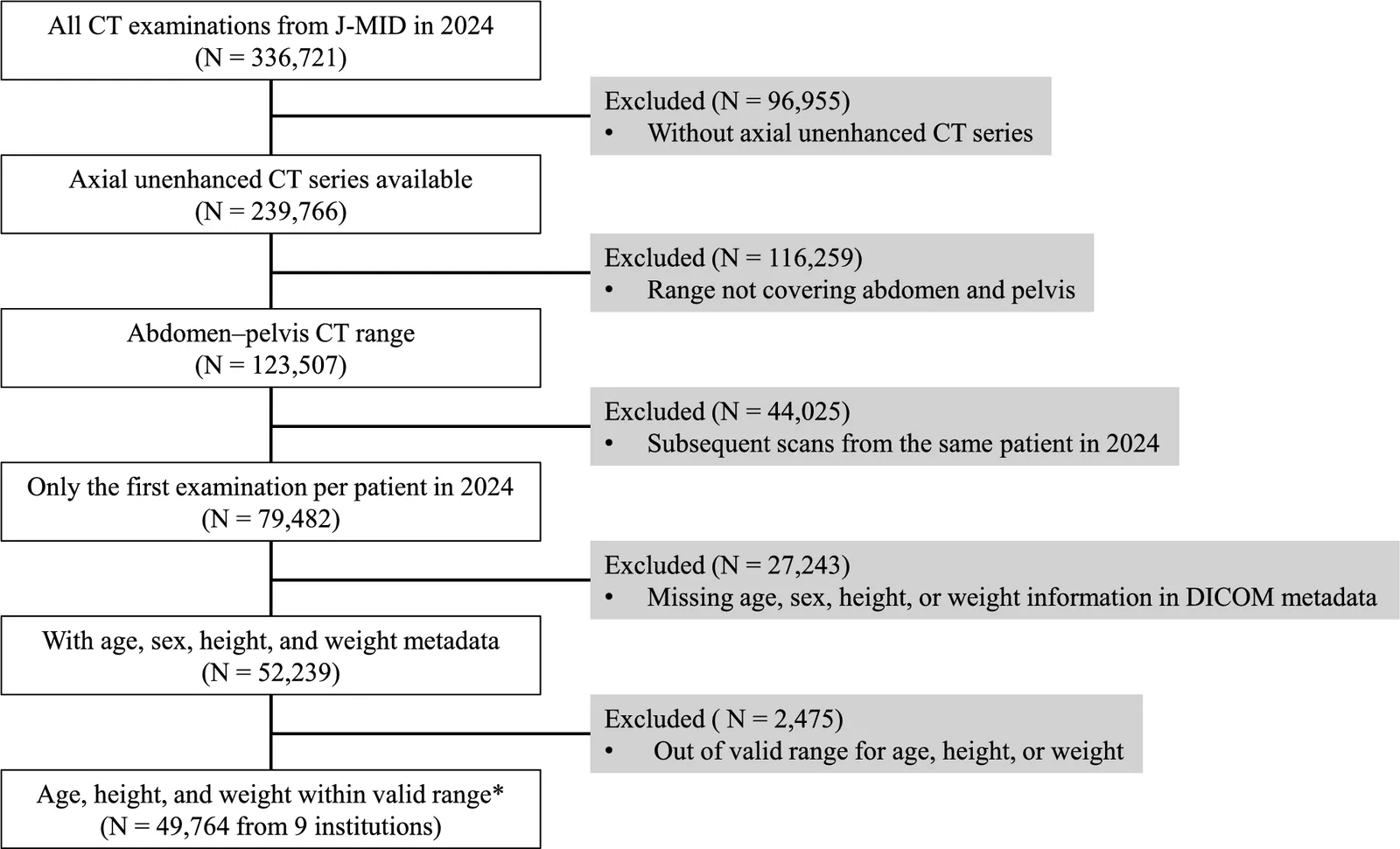
Flowchart of case selection. *Valid ranges were defined as age 20–89 years, height 1.0–2.0 m, and weight 30–120 kg

The patient selection flowchart used in this study is presented in Fig. 1. All abdominopelvic CT examinations performed in 2024 were screened for eligibility criteria. The inclusion criteria were as follows: (1) availability of an axial non-contrast series and (2) coverage of the abdomen and pelvis. Examinations were excluded if they met any of the following conditions: (1) multiple CT examinations obtained from the same patient within 2024 (only the first examination was included); (2) missing demographic or anthropometric information (age, sex, height, or weight) in the DICOM metadata; or (3) out-of-range anthropometric values (age < 20 or > 89 years, height < 1.0 or > 2.0 m, or weight < 30 or > 120 kg). These ranges were also applied to avoid the inclusion of outliers or potentially identifiable extreme values when publishing statistical information. After applying these criteria, 49,764 examinations were included in the final analyses.

### CT acquisition

The included CT examinations were acquired during routine clinical practice at the participating institutions of the J-MID. Scanner models, acquisition parameters, and reconstruction protocols varied among institutions and were not standardized.

### Image analysis

All the included CT datasets were processed using TotalSegmentator (version 2.10.0) for automated organ segmentation [14]. To make it computationally feasible to process the entire cohort on a single GPU, the processing was performed using the "--fast" option, which resamples input images to a 3-mm isotropic resolution prior to inference (rather than 1.5 mm in the standard mode), using third-order spline interpolation for image data. The processing pipeline was executed on a Linux workstation equipped with an NVIDIA RTX 6000 Ada GPU on Ubuntu 22.04. The abdominal and pelvic organs were extracted, whereas the hollow viscera were excluded from the TotalSegmentator output. The following organs were analyzed: liver, pancreas, spleen, right and left kidneys, right and left adrenal glands, prostate, and right and left iliopsoas muscles. The segmented volume was calculated for each organ using custom Python scripts. The performance of the TotalSegmentator was validated through a preliminary evaluation using a subset of our cohort (Online Appendix A). The potential effect of the "–fast" option on the resulting volume measurements was evaluated separately (Online Appendix B).

### Statistical analysis

Descriptive statistics, including age, height, weight, and body surface area (BSA), were initially calculated to summarize the characteristics of the study cohort by participating institutions and sex. The correlations between organ volume and demographic or anthropometric variables (age, sex, height, weight, and BSA) were preliminarily examined, and the results are presented in Online Appendix C. For each organ, sex-specific organ volumes were calculated as the 10th, 25th, 50th, 75th, and 90th percentiles based on the entire study cohort, excluding cases with zero-volume detection (treated as likely segmentation failures or organ absence).

**Fig. 2:**
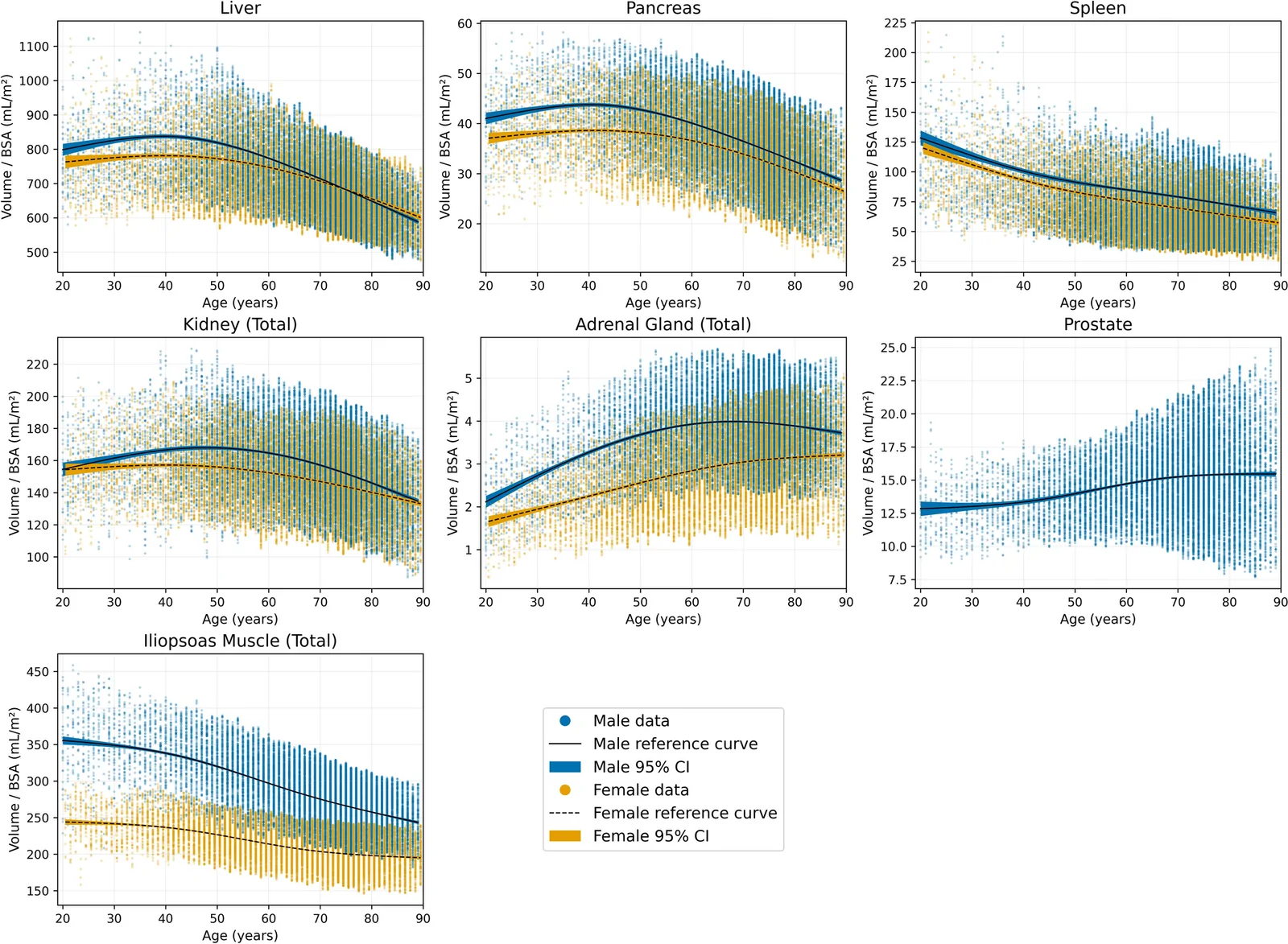
Relationship between age and body surface area–normalized organ volume. Scatterplots showing the relationship between age and organ volume normalized based on the body surface area in mL/m^2^ for each organ. Blue and orange dots represent male and female data points, respectively. Only data within the 10th–90th percentiles were plotted. The black solid and dashed lines indicate the smoothed reference curves generated using natural cubic spline functions with four degrees of freedom for males and females, respectively. Shaded bands around each curve indicate 95% confidence intervals

In addition, the relationship between age and organ volume normalized based on BSA (mL/m^2^) was visualized separately for males and females. Using all available data, smoothed reference curves were generated using natural cubic spline functions with four degrees of freedom, a choice consistent with general guidance that a small to moderate number of degrees of freedom typically provides an adequate balance between flexibility and stability in biomedical applications [15–17]. Sensitivity analyses fitting splines with three, six, and eight degrees of freedom are presented in Online Appendix D. Ninety-five percent confidence bands for each spline curve were computed and are displayed alongside the fitted curves in Fig. 2. The resulting age–volume relationships were visually classified into one of five patterns: higher volumes in older age groups, lower volumes in older age groups, convex (mountain-shaped), concave (valley-shaped), or flat (no apparent change). To safeguard participant privacy and minimize the risk of individual re-identification from uniquely small or large anatomical measurements, the tabulated results and scatter plots focused on the 10th–90th percentile range. This restriction was applied only to data presentation and visualization and did not affect the underlying statistical analyses, which were performed using the full dataset. No disease-specific or regional subgroup analyses were performed because detailed clinical and sociodemographic information was not uniformly available across institutions.

Sex differences in the age–volume relationships were tested on the BSA-normalized volumes (mL/m^2^) using nested-model F-tests on the sex × age (spline basis) interaction term. Three nested models were compared: a model with the spline of age only, a model adding a sex main effect, and a model adding the sex × age interaction. F-tests on the model differences provided p-values for the sex main effect and the sex × age interaction. As large sample sizes can produce small p-values for clinically negligible differences, effect sizes were also reported, defined as the maximum absolute and shape-related differences between fitted male and female curves across the age range.

All statistical analyses and plotting were performed using Python version 3.11.13, with the following packages: statsmodels 0.14.5, pandas 2.3.1, and matplotlib 3.10.5, along with their dependent libraries. Custom scripts used for data preprocessing and summarization are available from the corresponding author upon reasonable requests.

To assess the influence of organ-specific surgical history on the reference distributions, we performed a sensitivity analysis excluding cases with documented surgical history of the relevant organ. Details and results are provided in Online Appendix E.

## Results

In total, 49,764 CT examinations were included in the final analysis (26,456 men and 23,308 women). Of the 11 J-MID participating institutions, nine (A to I) provided eligible cases during the study period, with the number of cases per institution ranging from 171 to 10,491.

The basic demographic and anthropometric characteristics of the study cohort are presented in Table 1. Information on the CT manufacturer, pixel spacing, and slice thickness are summarized in Table 2. Toshiba scanners accounted for the largest proportion of examinations, followed by Canon, Siemens, GE, and Philips scanners. Although Toshiba Medical Systems has since merged with Canon Medical Systems, manufacturer categories were defined according to the original manufacturer information recorded in the DICOM headers to preserve consistency with the acquisition-time metadata.

**Table 1:** Demographic and anthropometric characteristics of the study cohort

	Male	Female
Sample size*	26,456 A:1311, B:3857, C:110, D:3790, E:4223, F:5536, G:3070, H:2945, I:1614	23,308 A:1322, B:2654, C:61, D:3441, E:3449, F:4955, G:2875, H:3199, I:1352
Age (year)	71 [60, 77]	67 [54, 76]
Height (m)	1.67 [1.63, 1.72]	1.55 [1.50, 1.59]
Weight (kg)	64.9 [57.5, 72.7]	52.3 [46.1, 60.0]
BSA (m^2^)**	1.73 [1.63, 1.84]	1.50 [1.40, 1.60]

All values except sample size are presented as median [25th, 75th percentile]

^*^ A–I indicate anonymized institution identifiers

^**^ Body Surface Area

**Table 2:** Distribution of CT information

Manufacturer	Toshiba	16,504
	Canon	14,938
	Siemens	8,730
	GE	6,849
	Philips	2,646
	Others or no information	97
Pixel spacing (mm)	> 1.0	3,648
	0.5 – 1.0	45,970
	< 0.5	146
Slice thickness (mm)	> = 7	15
	5 < = , < 7	38,814
	3 < = , < 5	10,743
	< 3	192

Sex-specific summaries of organ volumes are presented in Tables 3 and 4, respectively. For paired organs, such as the kidneys, adrenal glands, and iliopsoas muscles, the total volumes were calculated by summing the values for the right and left sides.

**Table 3:** Organ volumes in males

Organ	p10*	p25*	p50*	p75*	p90*
Liver (n = 26,456)	893.3	1024.4	1194.7	1411.4	1667.2
Pancreas (n = 26,432)	35.9	49.2	63.7	78.6	92.4
Spleen (n = 26,276)	58.6	83.6	118.7	168.0	234.8
Kidney right (n = 25,963)	89.7	111.3	134.1	159.5	188.1
Kidney left (n = 25,889)	89.7	114.6	139.0	165.1	193.0
Kidney total (n = 26,442)	178.0	221.9	268.3	317.2	369.2
Adrenal gland right (n = 26,182)	1.5	2.2	3.0	3.7	4.5
Adrenal gland left (n = 25,831)	1.9	2.9	3.8	4.7	5.6
Adrenal gland total (n = 26,377)	3.6	5.2	6.7	8.2	9.8
Prostate (n = 24,759)	15.7	20.6	25.5	30.8	36.8
Iliopsoas muscle right (n = 26,456)	166.8	199.9	237.6	279.3	323.2
Iliopsoas muscle left (n = 26,456)	170.2	205.5	246.2	290.5	334.5
Iliopsoas muscle total (n = 26,456)	339.6	406.5	483.4	568.0	655.1

The units of the values are milliliters (mL)

^*^p10, p25, p50, p75, and p90 indicate percentiles

**Table 4:** Organ volumes in females

Organ	p10*	p25*	p50*	p75*	p90*
Liver (n = 23,308)	776.1	886.0	1024.0	1203.9	1439.9
Pancreas (n = 23,266)	28.2	40.0	52.3	64.5	75.8
Spleen (n = 23,202)	48.7	67.8	95.4	134.3	187.4
Kidney right (n = 23,020)	75.0	91.5	109.1	129.0	151.8
Kidney left (n = 22,977)	76.5	96.1	115.1	135.6	158.5
Kidney total (n = 23,301)	150.8	185.6	221.2	260.0	302.6
Adrenal gland right (n = 22,773)	0.8	1.3	1.9	2.6	3.3
Adrenal gland left (n = 22,247)	1.0	1.7	2.5	3.3	4.1
Adrenal gland total (n = 23,100)	1.8	2.9	4.2	5.7	7.1
Iliopsoas muscle right (n = 23,308)	112.3	133.9	156.5	179.9	202.9
Iliopsoas muscle left (n = 23,308)	114.6	137.9	161.6	186.3	210.8
Iliopsoas muscle total (n = 23,308)	230.1	272.3	317.7	364.6	411.9

The units of the values are milliliters (mL)

^*^p10, p25, p50, p75, and p90 indicate percentiles

Figure 2 shows the relationship between age and body surface area–normalized organ volume (mL/m^2^) for each organ. Based on the visual inspection of the spline curves, the liver, pancreas, and kidneys exhibited similar convex patterns in both sexes, with largely overlapping distributions between males and females. In contrast, sex differences were more pronounced in the adrenal glands and the iliopsoas muscle. Adrenal gland volumes showed a convex pattern in males and a monotonic increase in females. Iliopsoas volumes were lower in older age groups in both sexes, with the pattern of decline differing between sexes. Prostate volumes in males demonstrated a wider range of values in older age groups, whereas spleen volumes declined with age and showed largely parallel curves between sexes. Online Appendix F provides summary tables of organ volumes according to BSA and age.

Formal sex × age interaction tests on the BSA-normalized volumes provided quantitative measures of these patterns (Table 5). The interaction was negligible for the spleen (shape-related difference 1.3% of median fitted volume; p = 0.93), modest for the liver, pancreas, and kidneys (shape-related differences 4–7%), and substantially larger for the adrenal glands and iliopsoas muscles (shape-related differences 13–16%).

**Table 5:** Formal sex × age interaction tests across organs

Organ	p (sex main effect)*	p (sex × age interaction)†	Shape diff %‡	Max diff %§
Liver	< 0.001	< 0.001	5.4	7.1
Pancreas	< 0.001	< 0.001	4.2	12.9
Spleen	< 0.001	0.93	1.3	11.7
Kidney right	< 0.001	< 0.001	6.5	10.2
Kidney left	< 0.001	< 0.001	5	7.6
Kidney total	< 0.001	< 0.001	5.2	8.2
Adrenal gland right	< 0.001	< 0.001	15.4	36
Adrenal gland left	< 0.001	< 0.001	13.4	34
Adrenal gland total	< 0.001	< 0.001	13.6	35.7
Iliopsoas muscle right	< 0.001	< 0.001	15.7	47.5
Iliopsoas muscle left	< 0.001	< 0.001	14.6	46.5
Iliopsoas muscle total	< 0.001	< 0.001	15.4	46.8

^*^p-value for the sex main effect from a nested-model comparison

^†^*p*-value for the sex × age interaction from a nested-model comparison

^‡^Maximum male–female difference in fitted volumes after centering (i.e., shape difference), expressed as a percentage of the median fitted volume

^§^Maximum male–female absolute difference in fitted volumes, expressed as a percentage of the median fitted volume

## Discussion

Using approximately 50,000 routine non-contrast abdominopelvic CT examinations from a nationwide, multi-institutional Japanese platform, we generated reference distributions and body surface area–normalized plots of organ volume versus age. Distinct cross-sectional age–volume relationships were observed, with convex patterns for the liver, pancreas, and kidneys in both sexes; monotonic declines for the spleen and iliopsoas in both sexes; and sex-specific patterns for the adrenal glands. Prostate volumes in males showed greater variability in the older age groups. Overall, given its sample size and analytical scope across age, sex, and body size, this study represents one of the most comprehensive abdominal CT volumetric analyses to date.

Previous CT-based organ volumetry studies have typically involved cohorts of several hundred to a few thousand participants and have provided estimates of “normal” organ volumes [7, 10, 11]. For example, Tamura et al. established normative spleen volume references in healthy Japanese adults using automated CT segmentation, and Kim et al. reported population-based and personalized reference intervals for liver and spleen volumes using CT and AI, demonstrating the clinical utility of population-specific reference modeling [10, 11]. However, these studies lacked sufficient scale to characterize empirical distributions stratified by age, sex, and body size. Despite the recent availability of large imaging datasets, few studies, particularly those from Western populations, support population-level reference distributions that integrate these factors [12, 19–21]. In this context, the present study represents one approach to addressing this gap through analyses based on nationwide Japanese CT data.

The age–volume relationships observed for the liver, pancreas, and kidneys are consistent with previously reported cross-sectional observations, including lower liver size and perfusion in older individuals, reduced kidney cortical volume after body-size adjustment, and peak pancreatic volume in early-to-mid adulthood with lower values in older age groups, particularly among patients with diabetes [8, 22, 23]. Adrenal volume showed age-associated and sex-specific differences consistent with prior studies based on several hundred cases, exhibiting a convex pattern in males and higher values in older age groups in females, underscoring the importance of sex-specific reference curves [9]. Spleen volume was modestly lower in older age groups and reflected body habitus, whereas skeletal muscle metrics, including psoas volume, showed age-related differences with marked sex effects [24, 25]. Together, these findings suggest that the cross-sectional reference curves are consistent with patterns reported in prior studies and are unlikely to be driven primarily by measurement artifacts. Prostate volumes were generally higher in older age groups, with greater variability after approximately 60 years of age, potentially reflecting benign prostatic hyperplasia and volume reduction following treatment [26]. The visual interpretation of the age–volume curves was broadly consistent with the formal sex × age interaction tests: organs with curves that appeared overlapping (e.g., spleen) corresponded to small interaction effect sizes, whereas organs with visibly different age-related patterns (adrenal glands and iliopsoas muscles) corresponded to substantially larger interaction effect sizes. We note that female pelvic organs (uterus, ovaries) could not be analyzed in the present study because they are not included in the predefined class set of TotalSegmentator. Inclusion of these organs using dedicated segmentation pipelines remains an important direction for future work to enable comprehensive sex-specific volumetric assessment alongside other abdominal organs.

The establishment of nationwide reference distributions has several important implications for clinical practice. The integration of deep learning–based segmentation with these references enables automated organ volume reporting in routine CT, with potential benefits for diagnostic accuracy and inter-observer consistency, as highlighted in recent reviews [27]. Beyond routine reporting, age- and body size–adjusted organ volume reference distributions may support investigations of imaging-based markers associated with age, identification of early or subclinical organ atrophy, and individualized risk stratification and longitudinal monitoring in precision medicine. By providing Asian-specific reference values, this study addresses ethnic and body-size differences underrepresented in Western-centric datasets, supporting equity in diagnostic imaging and age-related research. Although focused on Japan, these benchmarks may be applicable to other East Asian populations with similar body habitus and provide a basis for international comparison.

This study had several limitations. First, the cohort comprised individuals who underwent routine clinical imaging rather than healthy volunteers. Although not all abdominal organs are likely to be abnormal in the same individual, specific pathologies (e.g., cirrhosis or splenomegaly) may have introduced bias and could not be systematically excluded in the primary analysis because radiology reports were not uniformly available, although sensitivity analyses using report-based filtering showed minimal impact (Online Appendix E). Accordingly, these reference distributions reflect a routine clinical population rather than a strictly healthy cohort. Second, the reported values are specific to the segmentation pipeline used in this study (TotalSegmentator with the "–fast" option); comparisons with values obtained from future versions, other TotalSegmentator modes, or other segmentation models should therefore be made with caution (Online Appendix B). Although TotalSegmentator is a robust framework, segmentation errors may have influenced the aggregated data, as non-contrast CT can introduce measurement variability in structures with less clearly defined boundaries. Cases with zero-volume detection were excluded from the per-organ percentile calculations as likely segmentation failures or organ absence. To focus on the central distribution and reduce the re-identification risk, tabulated results and visualizations were limited to the 10th–90th percentile range. Variation in slice thickness may also have affected volume estimation, although this reflects the real-world clinical practice. Third, disease-specific and regional subgroup analyses were not performed because detailed clinical and sociodemographic data were not consistently available; these factors warrant further investigation in future studies. Fourth, the ethnic composition was unbalanced, as foreign residents accounted for approximately 3% of Japan’s population in 2024, and the dataset predominantly represented Japanese individuals [28]. Finally, this cross-sectional study captured population-level differences across age groups in 2024 and did not reflect longitudinal aging within individuals.

In conclusion, using a nationwide Japanese CT dataset, we established sex- and age-specific reference distributions for abdominal organ volume and identified cross-sectional age-associated differences. These CT-based references provide practical resources for clinical interpretation, epidemiology, and AI development.

## Supplementary Information

Below is the link to the electronic supplementary material.

Supplementary file1 (DOCX 1206 kb)

## Data Availability

The statistical data from this study will be shared upon reasonable request from the corresponding author. The original clinical data obtained from the J-MID cannot be publicly shared because of ethical and privacy restrictions.
