# Supplementary material for: Nationwide organ volume distributions and cross-sectional age-associated differences in abdominal CT from Japan
Source: Jpn J Radiol. 2026 Jun 11;44(10):1810–7. doi: 10.1007/s11604-026-02028-z (PMC13623781; doi:10.1007/s11604-026-02028-z)
Supplement: Supplementary file 1 [file 11604_2026_2028_MOESM1_ESM.pdf]

Appendix A. Preliminary evaluation of TotalSegmentator

A small-scale preliminary evaluation of automated organ segmentation was conducted using abdominopelvic CT examinations randomly sampled from the study cohort to form a sub-cohort. From the available data, 96 examinations were sampled from six institutions, with 16 cases per institution. For each case, segmentation results for the liver, spleen, pancreas, and bilateral kidneys generated by TotalSegmentator were compared with manually created reference labels generated by a single board-certified radiologist, and Dice similarity coefficients were calculated. Institutions in which 16 eligible examinations could not be secured on the same acquisition date were excluded from this preliminary analysis.

In addition to the five organs above, manual annotations of the bilateral adrenal glands and the prostate were performed on the same 96-case subset, and Dice similarity coefficients for these organs are reported below. Overall, the Dice similarity coefficients indicated acceptable segmentation performance across all eight organs, although the values for the adrenal glands were modestly lower, reflecting the small size and anatomical complexity of these organs.

**Organ**	**Facility**					
	**B**	**D**	**E**	**F**	**G**	**H**
Liver	0.970	0.974	0.976	0.977	0.979	0.971
Pancreas	0.854	0.885	0.891	0.890	0.901	0.887
Spleen	0.944	0.952	0.958	0.964	0.967	0.958
Kidney right	0.944	0.956	0.950	0.960	0.956	0.951
Kidney left	0.953	0.957	0.953	0.961	0.961	0.955
Adrenal gland right	0.873	0.855	0.865	0.853	0.839	0.852
Adrenal gland left	0.818	0.846	0.900	0.856	0.872	0.872
Prostate*	0.909	0.984	0.909	0.945	0.874	0.926

* The prostate was evaluated only in male cases. The number of male cases per institution was not balanced and was 4, 7, 10, 11, 7, and 7 for B, D, E, F, G, and H, respectively.

Appendix B. Sensitivity analysis: effect of the "–fast" option and slice thickness on volume measurements

B.1 Background

For computational tractability over a large cohort of 49,764 examinations, TotalSegmentator was run with the "–fast" option. As part of the internal pipeline of TotalSegmentator, input images were automatically resampled to a 3-mm isotropic resolution prior to inference, using third-order spline interpolation for image data, after which the output segmentation labels were resampled back to the native acquisition grid. Because the original slice thickness varied across the cohort, this resampling step has the potential to introduce systematic differences between "–fast" and standard (native-resolution) segmentation outputs, particularly for small organs.

To quantify the magnitude of these differences, we performed a paired comparison between the "–fast" and standard outputs of TotalSegmentator in a stratified subset of the study cohort.

B.2 Paired comparison design

A total of 200 examinations were sampled from the study cohort, comprising 100 cases at 3-mm slice thickness and 100 cases at 5-mm slice thickness. These two slice thicknesses were selected because they were the two most common slice thicknesses in the cohort and provided sufficient sample size for the comparison. For each case, segmentation was performed twice using the same version of TotalSegmentator (v2.10.0): once with the "–fast" option and once without. Per-organ volumes were computed from the resulting segmentation masks, and the median percentage difference (–fast minus standard, divided by the standard value) was calculated overall and stratified by slice thickness.

B.3 Results

Differences between the "–fast" and standard outputs were limited for most organs (typically within ±5%), with larger differences observed for the adrenal glands and the prostate. The detailed results are presented in Table B.1.

Table B.1. Median percentage difference between "–fast" and standard TotalSegmentator outputs (relative to the standard value), overall and stratified by original slice thickness.

Organ	Overall (n=200)	3-mm subset (n=100)	5-mm subset (n=100)
Liver	+1.70%	+1.05%	+2.17%
Pancreas	+4.87%	+4.58%	+4.99%
Spleen	+2.72%	+1.96%	+3.83%
Kidney right	+1.13%	+1.20%	+1.11%
Kidney left	+2.07%	+1.75%	+2.54%
Adrenal gland right	+4.27%	+6.70%	+0.63%
Adrenal gland left	+2.14%	+3.28%	+1.73%
Prostate	−4.85%	−5.61%	−2.64%

For most organs, the differences were small in absolute magnitude. For the adrenal glands and the prostate, which are small organs in which a few voxels can substantially affect the total volume, the differences were more pronounced. For several organs (liver, spleen, left kidney, pancreas), the differences were larger at 5-mm than at 3-mm slice thickness, suggesting that the systematic effect of the "–fast" resampling may be amplified as the original slice thickness increases beyond 5 mm. Cases with slice thickness exceeding 5 mm were rare in the present cohort and were therefore not analyzed separately.

B.4 Conclusion

The "–fast" option of TotalSegmentator introduced systematic but limited differences from standard (native-resolution) segmentation for most organs. Larger differences were observed for the adrenal glands and the prostate, and for some organs the differences were greater at 5-mm than at 3-mm slice thickness. Reference values for small organs such as the adrenal glands should therefore be interpreted in the context of the segmentation pipeline used in this study.

Appendix C. Correlation coefficients with organ volumes

Variable	Liver	Pancreas	Spleen	Kidney	Adrenal gland	Prostate	Iliopsoas muscle
Age	−0.322	−0.308	−0.198	−0.203	0.139	0.036	−0.258
Age^2	−0.340	−0.326	−0.196	−0.220	0.119	0.026	−0.263
Age^3	−0.348	−0.334	−0.191	−0.230	0.100	0.017	−0.263
Height	0.394	0.391	0.218	0.412	0.366	0.143	0.695
$Height^2$	0.395	0.392	0.219	0.413	0.367	0.142	0.698
$Height^3$	0.396	0.392	0.220	0.414	0.367	0.141	0.700
Weight	0.562	0.568	0.306	0.571	0.614	0.205	0.674
$Weight^2$	0.562	0.548	0.308	0.560	0.591	0.192	0.658
$Weight^3$	0.548	0.518	0.304	0.536	0.554	0.176	0.625
BSA	0.560	0.572	0.306	0.578	0.599	0.215	0.757
Sex (M)	0.233	0.271	0.148	0.297	0.448	—	0.634

* The row corresponding to BSA is shaded in gray. Red indicates the variable with the highest absolute correlation coefficient with organ volume, and green indicates those within the top three. For all organs analyzed, the absolute correlation coefficient for BSA ranked among the top three.

In addition, to examine the potential effect of bladder filling on prostate volume measurement, we examined the correlation between bladder volume and prostate volume in 24,757 male cases with both volumes available. The overall correlation was weak (Spearman $\rho = 0.22$), and within-decade correlations remained weak across age groups ($\rho = 0.07$–0.13 for ages 20–59; $\rho = 0.20$–0.28 for ages 70–89). Although several factors—including direct mechanical effects of bladder filling and age-related co-occurrence of bladder retention and benign prostatic hyperplasia—may contribute to this association, given the overall weak magnitude, no specific correction for bladder filling was applied in the present study.

Appendix D. Sensitivity analysis of spline degrees of freedom

To verify that the choice of four degrees of freedom for the spline visualization was not arbitrary, we fitted natural cubic splines with three, four, six, and eight degrees of freedom for each organ × sex combination and overlaid the resulting curves on the same axes (Figures D.1 to D.7). Shaded bands represent the 95% confidence intervals for each fitted curve.

Across the four values of degrees of freedom examined, curves at four, six, and eight degrees of freedom shared the same overall age-associated patterns. With three degrees of freedom, the curves were noticeably stiffer for some organs and missed features that appeared consistent with the underlying data, particularly for the iliopsoas muscles. With six or eight degrees of freedom, additional fluctuations were observed mainly in age regions with smaller sample sizes (notably the youngest age groups). These additional fluctuations are more plausibly attributable to overfitting of local sampling variability than to biological signal. Four degrees of freedom were therefore retained as the visualization choice.

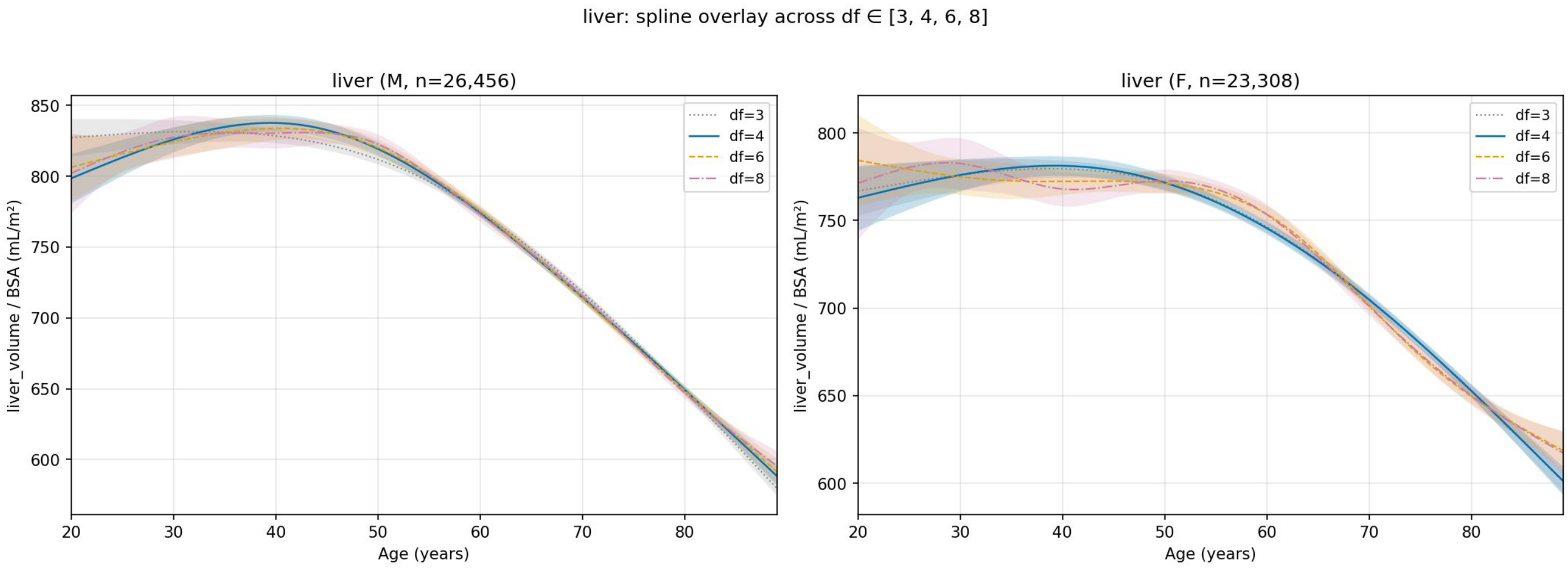

Figure D.1. Liver: spline overlay across df ∈ {3, 4, 6, 8} for males and females.

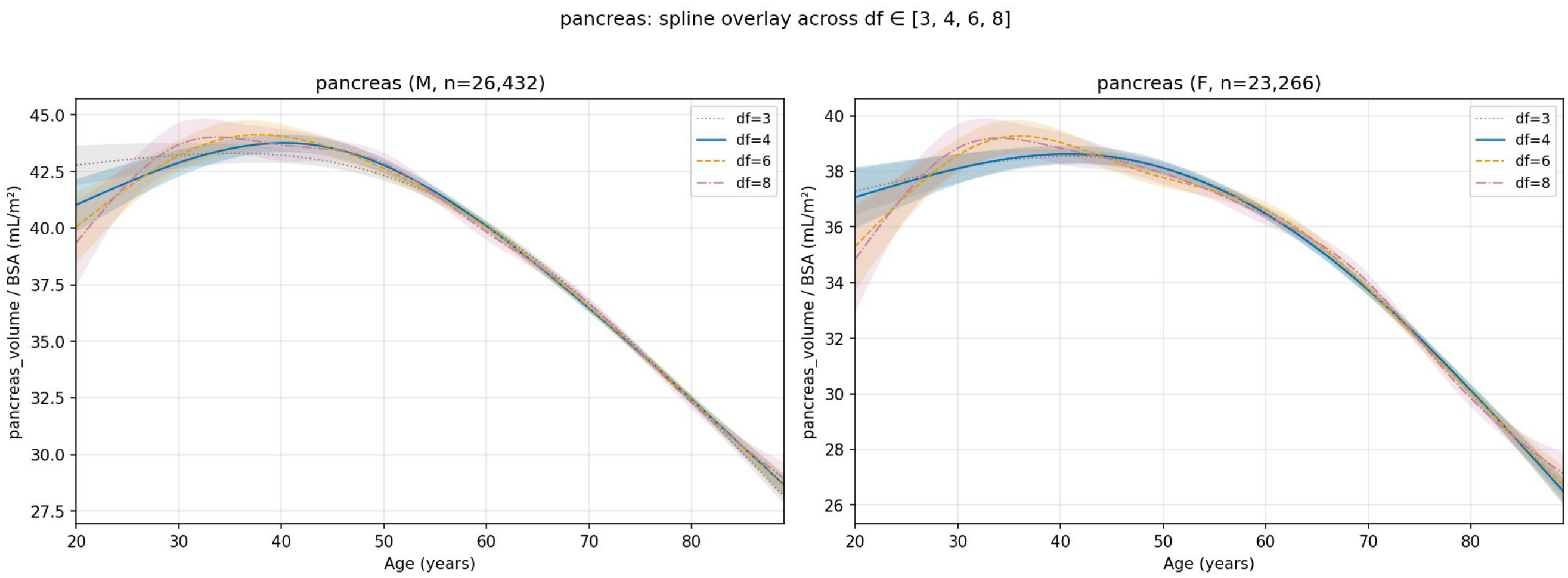

Figure D.2. Pancreas: spline overlay across df ∈ {3, 4, 6, 8} for males and females.

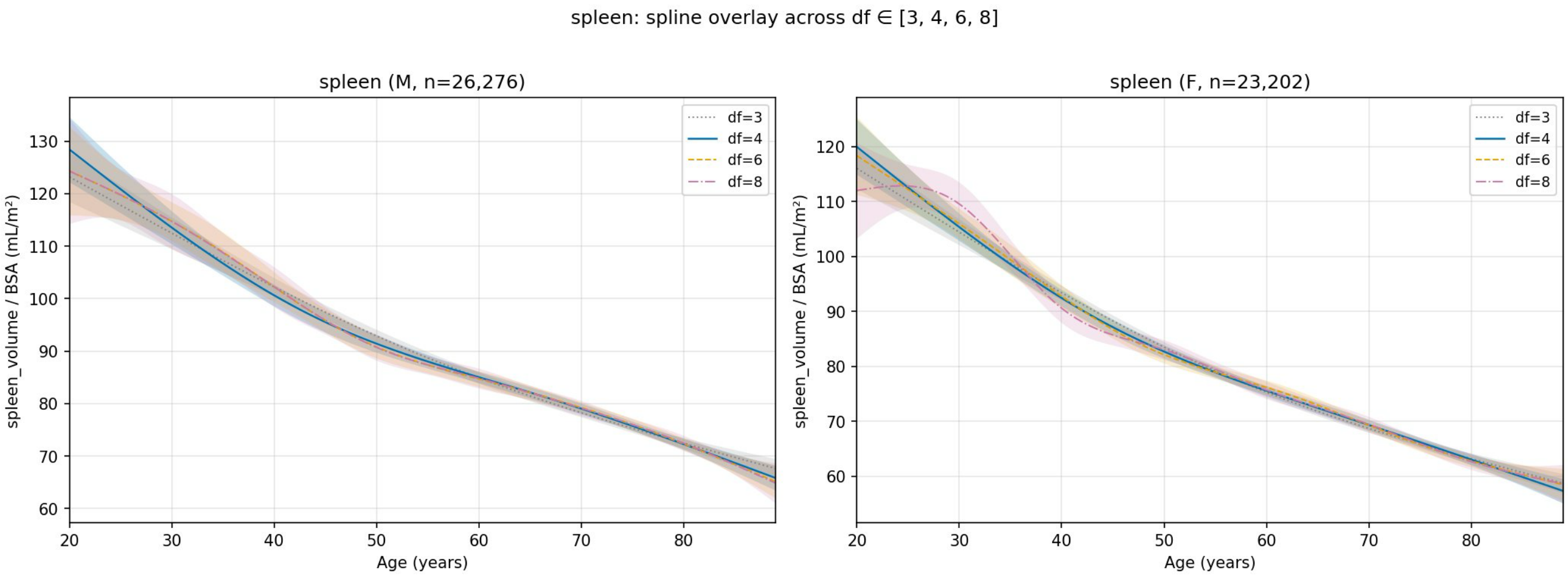

Figure D.3. Spleen: spline overlay across df ∈ {3, 4, 6, 8} for males and females.

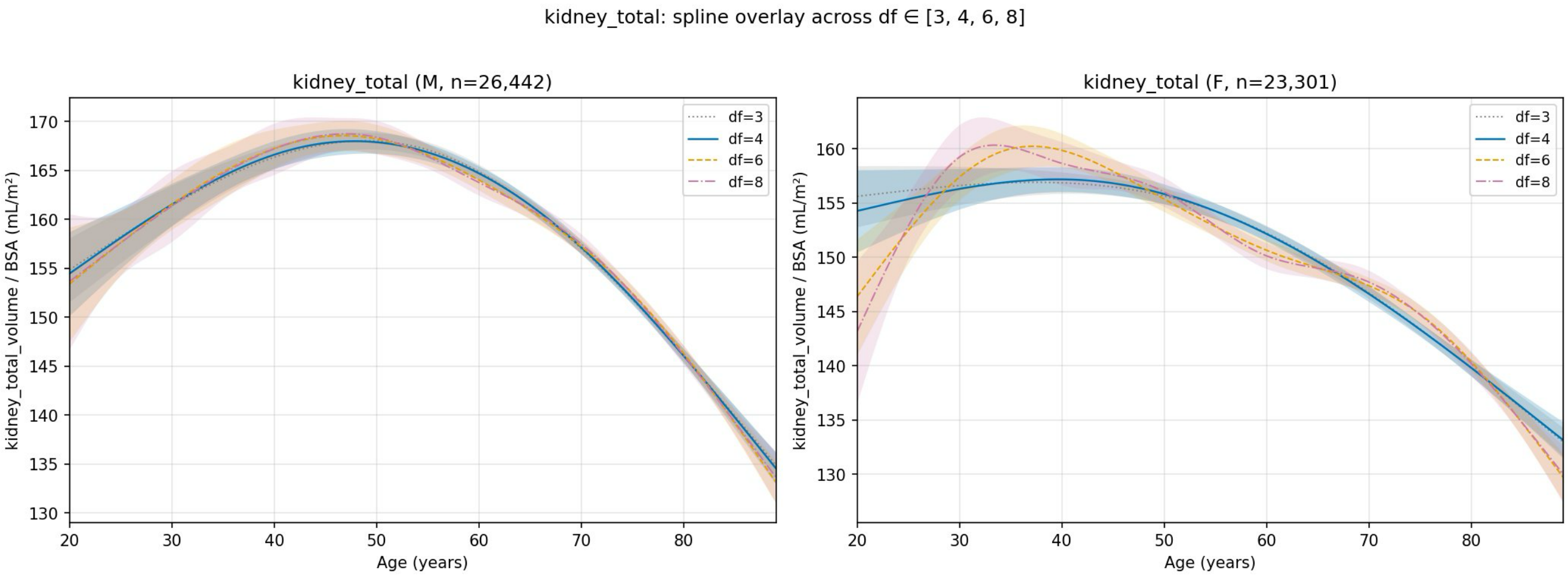

Figure D.4. Kidney total: spline overlay across df ∈ {3, 4, 6, 8} for males and females.

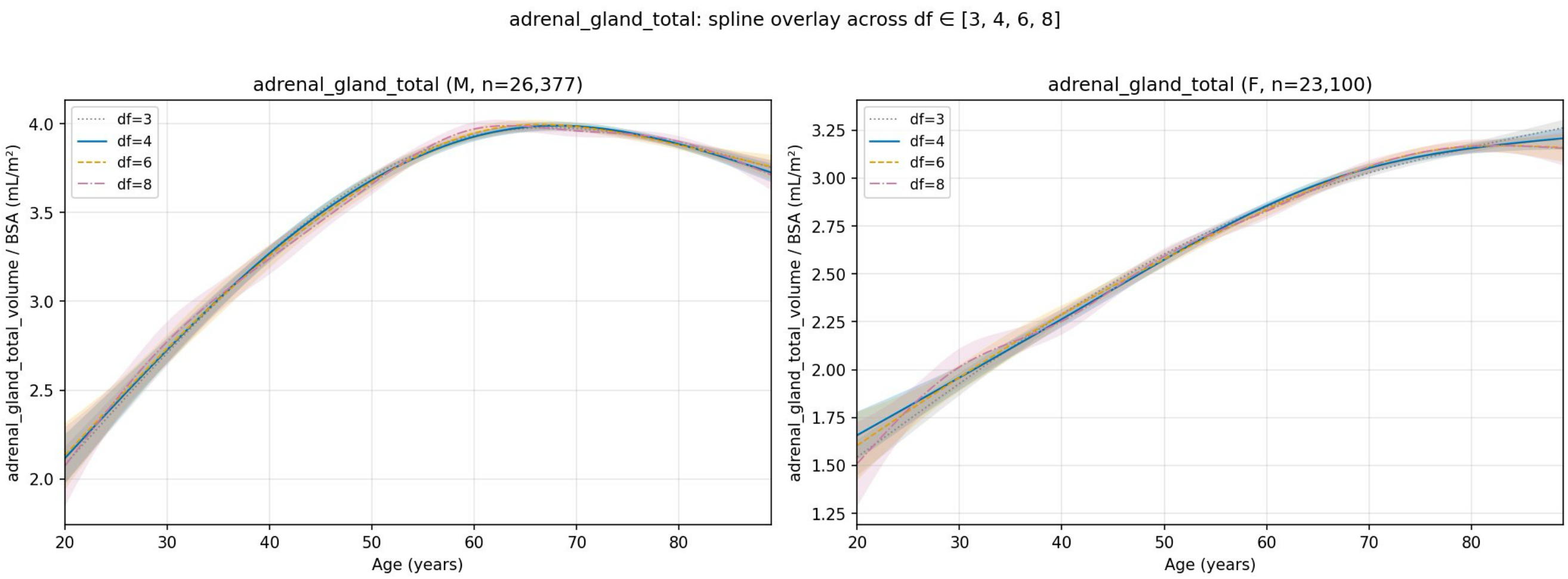

Figure D.5. Adrenal gland total: spline overlay across df ∈ {3, 4, 6, 8} for males and females.

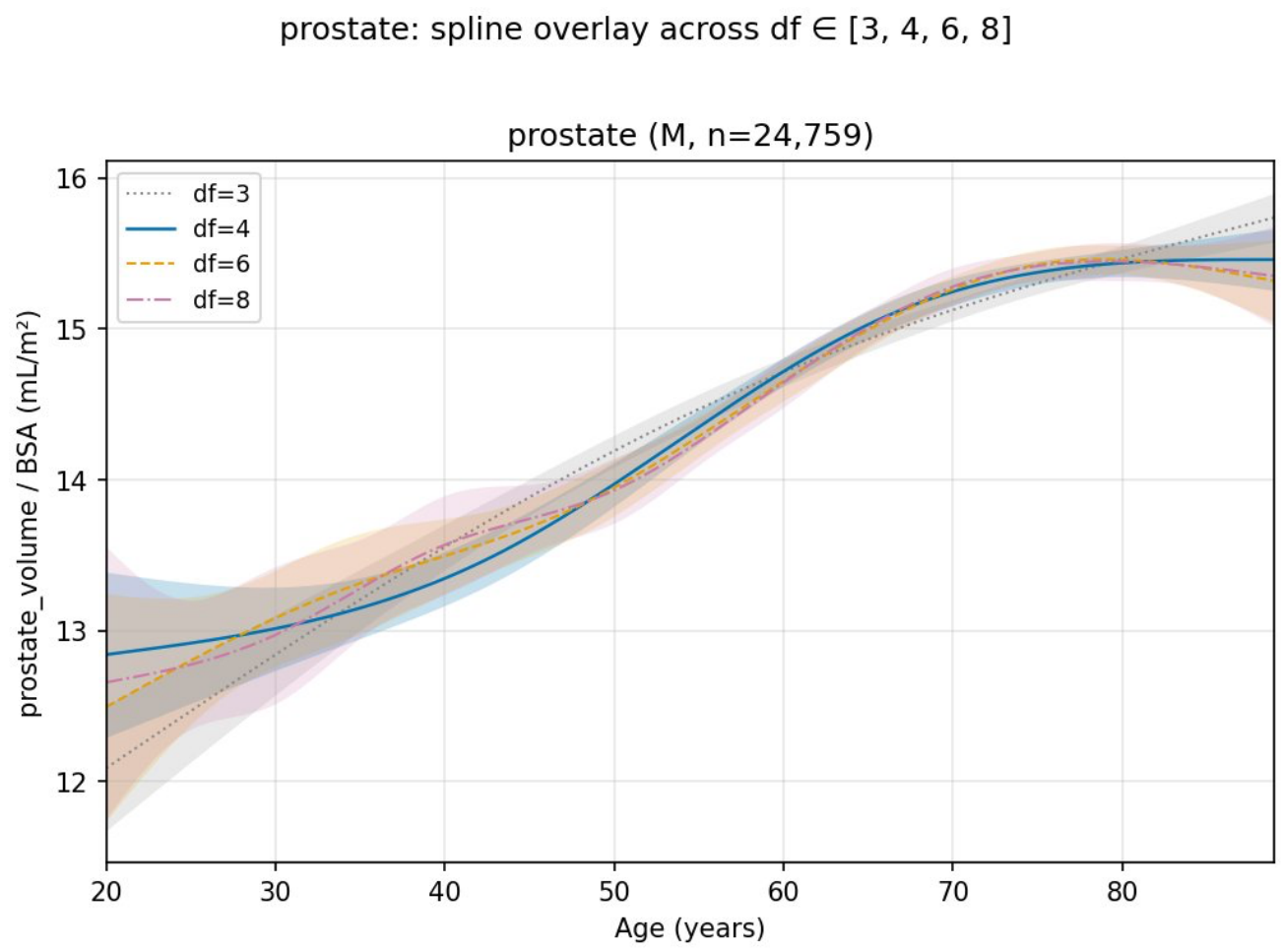

Figure D.6. Prostate: spline overlay across df ∈ {3, 4, 6, 8} for males.

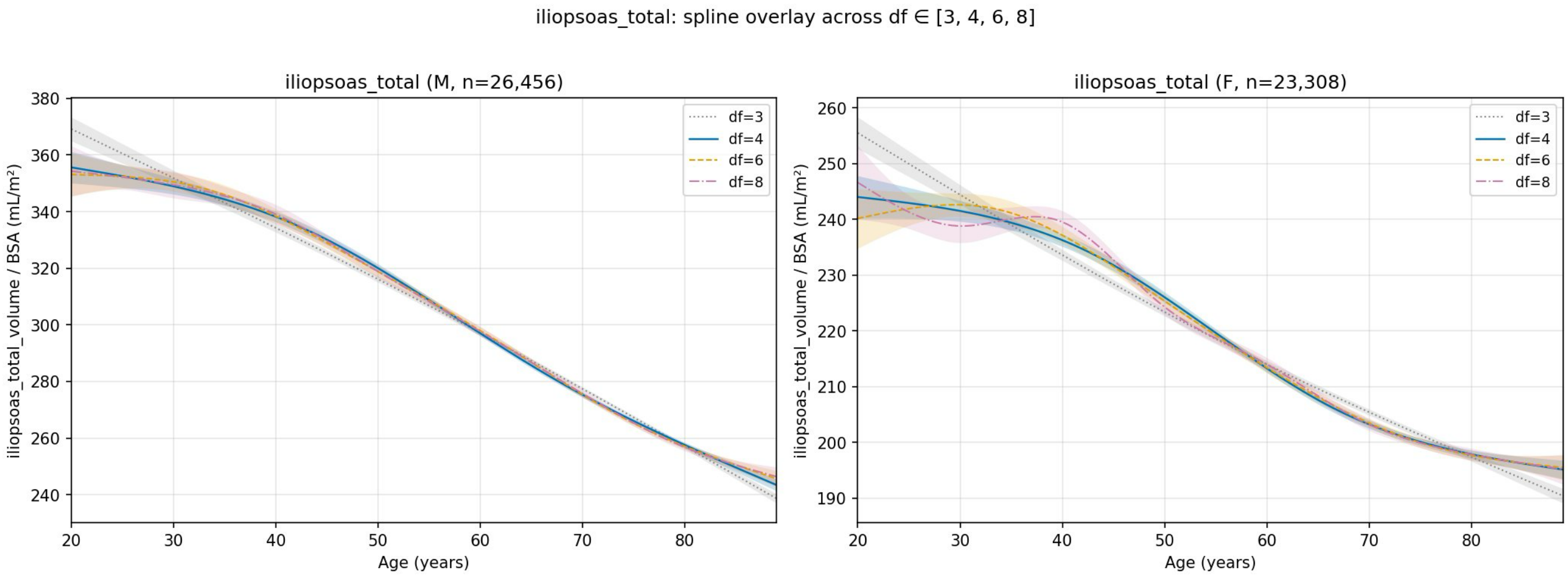

Figure D.7. Iliopsoas muscle total: spline overlay across df ∈ {3, 4, 6, 8} for males and females.

Appendix E. Sensitivity analysis: exclusion of cases with documented organ-specific surgical history

E.1 Identification of surgical history from radiology reports

Of the 49,764 examinations included in the primary analysis, **34,475 (69.3%) had an associated radiology report** that could be queried for surgical history. The remaining 15,289 (30.7%) examinations had no available report and were therefore not classifiable for surgical history; these cases were retained in the primary analysis but excluded from the sensitivity analysis.

For the 34,475 examinations with available reports, surgical history of the eight target organs (liver, pancreas, spleen, right and left kidneys, right and left adrenal glands, and prostate) was extracted using a locally hosted large language model (gpt-oss-20b) with a structured precision-oriented prompt (the full prompt is provided in Section E.5). To validate the classification, 40 randomly selected cases per organ category (20 predicted positive and 20 predicted negative; 240 cases total) were manually reviewed by a board-certified radiologist. Precision was 1.00 for all organs, and recall ranged from 0.95 to 1.00.

The number of cases with documented surgical history of each organ within the report-available cohort (n = 34,475) is summarized below.

Organ	Cases with documented surgery	% of report-available cohort
Liver	934	2.71%
Pancreas	595	1.73%
Spleen	231	0.67%
Kidney right	1,329	3.85%
Kidney left	1,472	4.27%
Adrenal gland right	103	0.30%
Adrenal gland left	110	0.32%
Prostate (males only, n = 18,331)	637	3.47%

For paired organs, surgery on either side was documented in 2,368 cases (6.87%) for the kidneys and 193 cases (0.56%) for the adrenal glands.

E.2 Exclusion logic

For each organ, cases with documented surgical history of that organ were excluded from the sensitivity analysis. For paired organs (kidneys and adrenal glands), cases with surgical history on either side were excluded from the analyses of both sides. The 15,289 cases without an available radiology report were also excluded from the sensitivity analysis, as their surgical history could not be ascertained.

E.3 Sensitivity analysis results

After applying the exclusion logic above, percentiles (P10, P25, P50, P75, P90) were recomputed by sex and organ and compared with the primary analysis (Tables E.1, E.2). Across all 125 cells (sex × organ × percentile), the maximum absolute difference between the primary and sensitivity analyses was **+5.66%** (prostate P10), followed by total kidney P10 in males (+4.75%), right adrenal P10 in females (−3.45%), pancreas P10 in females (+3.45%), and left kidney P10 in males (+3.77%). The

differences in median (P50) were within ±1.5% for all organs, and within the interquartile range (P25, P50, P75) the maximum difference was +2.62% (prostate P25).

Table E.1. Sensitivity analysis results in males. Percentile differences (Δ%) between the primary and sensitivity analyses (sensitivity analysis: cases with available reports and no documented surgery on the relevant organ).

Organ	n (Main)	n (Sens)	P10 Δ%	P25 Δ%	P50 Δ%	P75 Δ%	P90 Δ%
Liver	26,456	17,753	+0.42	+0.40	+0.32	+0.25	+0.19
Pancreas	26,432	17,967	+3.01	+1.81	+1.29	+0.84	+0.32
Spleen	26,276	18,132	+0.09	−0.81	−0.93	−0.40	−1.48
Kidney right	25,963	16,657	+1.20	+0.30	−0.52	−0.92	−1.21
Kidney left	25,889	16,644	+3.77	+0.75	+0.02	−0.74	−1.15
Kidney total	26,442	16,738	+4.75	+2.51	+1.10	+0.50	+0.29
Adrenal gland right	26,182	18,038	−1.75	+0.00	−0.91	−0.73	−0.60
Adrenal gland left	25,831	17,781	+0.00	+0.00	+0.00	+0.00	−0.48
Adrenal gland total	26,377	18,172	−0.75	−1.05	−0.81	−0.66	−0.28
Prostate	24,759	16,601	+5.66	+2.62	+1.48	+1.05	+0.88
Iliopsoas muscle right	26,456	18,331	+0.22	+0.16	+0.37	+0.25	+0.31
Iliopsoas muscle left	26,456	18,331	+0.06	+0.29	+0.59	+0.42	+0.52
Iliopsoas muscle total	26,456	18,331	+0.02	+0.29	+0.44	+0.32	+0.50

Table E.2. Sensitivity analysis results in females. Percentile differences (Δ%) between the primary and sensitivity analyses (sensitivity analysis: cases with available reports and no documented surgery on the relevant organ).

Organ	n (Main)	n (Sens)	P10 Δ%	P25 Δ%	P50 Δ%	P75 Δ%	P90 Δ%
Liver	23,308	15,788	+0.45	+0.37	+0.16	+0.09	+0.86
Pancreas	23,266	15,870	+3.45	+1.69	+0.77	+0.38	+0.43
Spleen	23,202	16,005	+0.33	−0.20	−0.85	−0.68	−0.13
Kidney right	23,020	15,293	−0.03	−0.44	−0.87	−0.90	−0.57
Kidney left	22,977	15,272	+1.31	+0.20	−0.45	−0.56	−0.48
Kidney total	23,301	15,359	+2.73	+0.92	+0.05	+0.05	+0.29
Adrenal gland right	22,773	15,667	−3.45	−2.13	+0.00	−1.03	+0.00
Adrenal gland left	22,247	15,299	+2.78	+1.61	+0.00	+0.00	−0.66
Adrenal gland total	23,100	15,891	+0.00	+0.00	+0.00	−0.47	−0.38
Prostate	23,308	16,144	−0.47	−0.02	−0.21	−0.02	+0.07
Iliopsoas muscle right	23,308	16,144	−0.70	−0.20	−0.02	−0.01	+0.05
Iliopsoas muscle left	23,308	16,144	−0.77	−0.12	−0.05	−0.07	+0.06

E.4 Conclusion

Excluding cases with documented organ-specific surgical history and cases without available reports from the primary cohort had a minimal effect on the reference distributions. The maximum percentile difference was +5.66% (prostate P10), and within the interquartile range (P25, P50, P75) the maximum difference was +2.62%. The reference distributions presented in the main text are therefore robust to potential bias introduced by the inclusion of postoperative cases.

E.5 LLM prompt for surgical history extraction

The following Japanese-language prompt was used with the locally hosted large language model (gpt-oss-20b) to extract organ-specific surgical history from radiology reports. The Japanese version below was the actual prompt fed to the model; an English translation is provided afterward for reference.

あなたは日本語の放射線レポートから対象臓器の手術歴の有無を判定する分類器です。各臓器について、その臓器に対する外科的切除・摘出・移植などの記載があるかを判定してください。

対象臓器（8 項目）：
- liver（肝臓）
- pancreas（膵臓）
- spleen（脾臓）
- right_kidney（右腎）
- left_kidney（左腎）
- right_adrenal（右副腎）
- left_adrenal（左副腎）
- prostate（前立腺）

判定：
- 1: 当該臓器に対する手術歴の明確な記載あり（部分切除・全摘・移植など臓器体積に影響する外科的介入すべて）
- 0: 手術歴の記載なし、判別不能、または記載が曖昧な場合

判定ルール：
1. 明確な記載がある場合のみ 1。記載がなければ 0 がデフォルト。
2. 両側臓器（腎・副腎）は左右別に判定する：
   - 「右腎摘後」→ right_kidney: 1, left_kidney: 0
   - 「腎摘後」のみで側方未記載 → right_kidney: 1, left_kidney: 1（判別不能ではあるが両方除外）
3. 移植はその臓器名を 1 にする「腎移植後」→ right_kidney: 1, left_kidney: 1
4. 「術後」とのみ記載され臓器が特定できない場合は 0。
5. 迷ったら 0（precision を優先）。

出力形式：
出力は JSON オブジェクトのみ。前置き・コードフェンス・説明文は一切付けない。

例：
レポート: 右腎摘出後の状態。残存左腎に有意な異常を認めない。肝・脾・膵に明らかな異常なし。
出力: {"liver":0,"pancreas":0,"spleen":0,"right_kidney":1,"left_kidney":0,"right_adrenal":0,"left_adrenal":0,"prostate":0}

レポート: 肝 S5 に径 15mm の低吸収結節を認める。脾腫あり。膵に明らかな異常なし。両腎に石灰化を散見。
出力: {"liver":0,"pancreas":0,"spleen":0,"right_kidney":0,"left_kidney":0,"right_adrenal":0,"left_adrenal":0,"prostate":0}

レポート: 前立腺全摘後。膀胱底部に術後変化あり。リンパ節腫大なし。
出力: {"liver":0,"pancreas":0,"spleen":0,"right_kidney":0,"left_kidney":0,"right_adrenal":0,"left_adrenal":0,"prostate":1}

レポート: 肝右葉切除後。残肝に再発疑い病変なし。脾摘後。
出力: {"liver":1,"pancreas":0,"spleen":1,"right_kidney":0,"left_kidney":0,"right_adrenal":0,"left_adrenal":0,"prostate":0}

レポート: 膵頭十二指腸切除後。残膵に異常所見なし。
出力: {"liver":0,"pancreas":1,"spleen":0,"right_kidney":0,"left_kidney":0,"right_adrenal":0,"left_adrenal":0,"prostate":0}

レポート: 腎移植後。右腸骨窩に移植腎を認める。両側ネイティブ腎は萎縮性。
出力: {"liver":0,"pancreas":0,"spleen":0,"right_kidney":1,"left_kidney":1,"right_adrenal":0,"left_adrenal":0,"prostate":0}

English translation (for reference; the Japanese version above was used in the actual run):

```
You are a classifier that determines, from a Japanese radiology report, whether documented surg
ical history exists for each target organ. For each organ, judge whether the report contains do
cumentation of surgical resection, removal, transplantation, or similar interventions.

Target organs (8 items):
- liver
- pancreas
- spleen
- right_kidney
- left_kidney
- right_adrenal
- left_adrenal
- prostate

Labels:
- 1: A clear description of surgical history of the organ is present (including any surgical in
tervention affecting organ volume, such as partial resection, total resection, or transplantati
on).
- 0: No description of surgical history, indeterminate, or ambiguous documentation.

Classification rules:
1. Assign 1 only when a clear description exists; absent any description, default to 0.
2. For paired organs (kidneys, adrenal glands), classify the right and left sides separately:
   - "Status post right nephrectomy" → right_kidney: 1, left_kidney: 0
   - "Status post nephrectomy" without side specification → right_kidney: 1, left_kidney: 1 (in
determinate, but both excluded)
3. For transplantation, set the relevant organ to 1: "Status post kidney transplantation" → rig
ht_kidney: 1, left_kidney: 1
4. If only "postoperative" is mentioned without specifying the organ, assign 0.
5. When in doubt, assign 0 (prioritize precision).

Output format:
Output the JSON object only. Do not include any preamble, code fences, or explanatory text.

Examples:
Report: Status post right nephrectomy. No significant abnormality in the remnant left kidney. N
o apparent abnormality in the liver, spleen, or pancreas.
Output: {"liver":0,"pancreas":0,"spleen":0,"right_kidney":1,"left_kidney":0,"right_adrenal":0,"
left_adrenal":0,"prostate":0}

Report: A 15-mm low-attenuation nodule is noted in liver segment 5. Splenomegaly is present. No
 apparent pancreatic abnormality. Scattered calcifications are seen in both kidneys.
Output: {"liver":0,"pancreas":0,"spleen":0,"right_kidney":0,"left_kidney":0,"right_adrenal":0,"
left_adrenal":0,"prostate":0}

Report: Status post total prostatectomy. Postoperative changes are noted at the bladder base. N
o lymphadenopathy.
Output: {"liver":0,"pancreas":0,"spleen":0,"right_kidney":0,"left_kidney":0,"right_adrenal":0,"
left_adrenal":0,"prostate":1}

Report: Status post right hepatectomy. No suspicious recurrent lesion in the remnant liver. Sta
tus post splenectomy.
Output: {"liver":1,"pancreas":0,"spleen":1,"right_kidney":0,"left_kidney":0,"right_adrenal":0,"
left_adrenal":0,"prostate":0}

Report: Status post pancreaticoduodenectomy. No abnormal findings in the remnant pancreas.
Output: {"liver":0,"pancreas":1,"spleen":0,"right_kidney":0,"left_kidney":0,"right_adrenal":0,"
left_adrenal":0,"prostate":0}

Report: Status post kidney transplantation. The transplanted kidney is identified in the right
iliac fossa. Both native kidneys are atrophic.
Output: {"liver":0,"pancreas":0,"spleen":0,"right_kidney":1,"left_kidney":1,"right_adrenal":0,"
left_adrenal":0,"prostate":0}
```

Appendix F. Summary tables by age and body surface area

The table presents median values and the 25th and 75th percentiles in milliliters (ml), with age on the vertical axis and body surface area (BSA) on the horizontal axis. Cells with insufficient data (n <10) are left empty.

Liver (Male)

Age (years) \ BSA (m^2)	1.3-1.4	1.4-1.5	1.5-1.6	1.6-1.7	1.7-1.8	1.8-1.9	1.9-2.0	2.0-2.1	2.1-2.2	2.2-2.3
**20-24**		1109.0 [951.9, 1274.8]	1151.2 [1040.7, 1308.8]	1242.6 [1143.2, 1405.1]	1294.9 [1190.5, 1416.8]	1453.1 [1322.9, 1561.9]	1609.0 [1490.2, 1748.3]			
**25-29**			1148.1 [965.6, 1277.3]	1224.5 [1108.2, 1363.5]	1347.6 [1261.5, 1505.1]	1517.9 [1359.7, 1628.9]	1607.1 [1314.8, 1726.1]	1669.2 [1438.6, 1861.1]		
**30-34**			1168.6 [1069.7, 1280.9]	1414.7 [1230.9, 1521.4]	1385.9 [1242.1, 1518.5]	1411.9 [1249.3, 1638.8]	1561.5 [1397.7, 1806.6]	1686.5 [1494.4, 1896.2]	1839.9 [1726.6, 2066.0]	
**35-39**			1261.7 [1069.7, 1388.3]	1317.6 [1133.0, 1521.6]	1336.6 [1223.5, 1522.5]	1510.7 [1396.3, 1656.8]	1556.5 [1401.7, 1758.9]	1667.2 [1544.2, 1954.9]	1954.6 [1830.5, 2230.7]	
**40-44**		1147.8 [1031.0, 1243.5]	1169.0 [1058.0, 1376.7]	1219.0 [1121.7, 1413.0]	1329.0 [1207.0, 1496.1]	1477.6 [1331.0, 1724.4]	1622.3 [1427.1, 1833.1]	1769.0 [1514.0, 1955.9]	1888.4 [1603.2, 2167.0]	
**45-49**		1040.5 [934.9, 1174.0]	1139.8 [1056.3, 1230.1]	1247.7 [1113.4, 1448.8]	1354.8 [1217.6, 1513.8]	1414.0 [1285.6, 1652.5]	1578.9 [1385.6, 1801.9]	1714.5 [1467.9, 1918.8]	1805.0 [1676.4, 2220.8]	2017.0 [1805.2, 2301.6]
**50-54**		1032.9 [838.8, 1155.7]	1176.4 [1021.4, 1334.6]	1278.6 [1140.3, 1425.2]	1335.0 [1190.5, 1553.2]	1434.1 [1265.2, 1644.8]	1592.4 [1388.8, 1778.2]	1767.6 [1576.9, 1982.0]	1925.6 [1697.1, 2163.5]	1950.6 [1718.5, 2319.7]
**55-59**		1001.1 [859.5, 1100.0]	1125.3 [998.7, 1296.0]	1206.3 [1101.4, 1373.5]	1347.4 [1199.4, 1498.3]	1404.9 [1263.2, 1564.9]	1517.4 [1357.2, 1766.8]	1638.7 [1489.6, 1857.2]	1691.0 [1468.1, 1965.6]	1853.2 [1657.2, 2004.3]
**60-64**	896.0 [836.4, 930.7]	1025.2 [923.2, 1129.7]	1068.4 [983.9, 1215.4]	1176.8 [1066.1, 1325.4]	1306.2 [1143.0, 1455.9]	1394.6 [1249.9, 1573.8]	1489.0 [1321.4, 1672.0]	1552.6 [1369.4, 1778.3]	1691.5 [1471.5, 1956.4]	1852.0 [1641.7, 2488.5]
**65-69**	938.9 [839.5, 1045.3]	993.5 [881.9, 1112.1]	1078.1 [961.5, 1198.9]	1143.8 [1041.1, 1261.0]	1240.1 [1118.7, 1407.2]	1323.4 [1194.0, 1494.3]	1440.0 [1281.0, 1625.9]	1529.1 [1367.6, 1707.0]	1665.3 [1408.8, 1965.7]	1831.5 [1724.6, 2002.5]
**70-74**	857.9 [751.6, 996.2]	972.5 [858.5, 1081.4]	1034.5 [934.1, 1159.4]	1109.7 [997.3, 1242.5]	1196.0 [1068.6, 1345.3]	1287.4 [1162.8, 1435.5]	1394.0 [1238.3, 1568.6]	1472.2 [1294.1, 1689.3]	1482.8 [1305.0, 1763.1]	
**75-79**	866.1 [748.4, 960.1]	923.5 [831.8, 1043.6]	995.2 [885.9, 1116.5]	1072.8 [958.5, 1200.3]	1142.9 [1023.8, 1285.2]	1229.6 [1112.9, 1398.3]	1282.5 [1169.6, 1452.4]	1411.3 [1270.3, 1567.6]	1541.9 [1440.5, 1656.2]	
**80-84**	797.9 [695.8, 927.6]	891.4 [796.5, 1000.2]	959.9 [862.0, 1060.6]	1028.5 [918.6, 1146.1]	1102.2 [995.3, 1227.4]	1144.3 [1036.0, 1264.0]	1230.5 [1103.8, 1417.6]	1301.8 [1197.8, 1567.1]		
**85-89**	821.3 [714.1, 886.2]	881.7 [775.4, 957.3]	932.4 [836.5, 1021.7]	990.5 [894.4, 1097.6]	1071.8 [954.2, 1188.3]	1128.6 [1029.1, 1263.0]	1237.7 [1073.7, 1378.5]			

Liver (Female)

Age (years) \ BSA (m^2)	1.1-1.2	1.2-1.3	1.3-1.4	1.4-1.5	1.5-1.6	1.6-1.7	1.7-1.8	1.8-1.9	1.9-2.0	2.0-2.1
**20-24**		904.2 [812.7, 939.6]	1024.3 [949.1, 1140.6]	1025.2 [927.5, 1147.0]	1122.8 [1010.6, 1279.8]	1184.6 [1046.8, 1449.4]	1253.3 [1156.5, 1446.2]			
**25-29**		925.2 [812.4, 1011.2]	989.0 [904.5, 1064.3]	1061.4 [940.4, 1172.2]	1097.3 [999.1, 1274.8]	1283.6 [1084.4, 1427.0]	1513.3 [1204.8, 1893.9]	1677.3 [1371.1, 1767.6]		
**30-34**		909.8 [840.2, 1032.6]	974.8 [878.1, 1147.8]	1053.4 [971.6, 1180.9]	1143.1 [1020.9, 1288.8]	1275.9 [1134.9, 1487.0]	1332.1 [1234.9, 1495.4]	1413.5 [1237.8, 1661.9]	2094.9 [1946.6, 2220.2]	
**35-39**		962.3 [880.2, 1019.8]	944.2 [892.9, 1052.1]	1049.4 [960.5, 1171.8]	1104.6 [986.3, 1261.4]	1259.3 [1136.1, 1357.5]	1304.4 [1182.6, 1518.6]	1477.9 [1305.7, 1655.9]	1592.5 [1509.1, 1983.1]	
**40-44**		865.1 [773.7, 991.1]	987.3 [860.5, 1098.8]	1039.1 [929.4, 1149.5]	1118.2 [1024.4, 1252.0]	1230.3 [1105.2, 1350.9]	1372.0 [1224.3, 1509.7]	1668.9 [1438.1, 1841.1]	1546.5 [1433.4, 1793.0]	1953.3 [1725.3, 2177.2]
**45-49**		888.2 [773.4, 1052.5]	975.1 [863.4, 1083.6]	1027.1 [930.0, 1154.5]	1125.6 [1014.9, 1266.6]	1222.2 [1088.2, 1404.8]	1324.0 [1174.9, 1506.7]	1570.1 [1325.0, 1755.1]	1659.9 [1526.7, 1925.6]	1941.8 [1571.6, 2068.7]
**50-54**		900.8 [808.4, 1012.1]	969.3 [864.0, 1062.9]	1026.0 [924.8, 1167.6]	1097.5 [995.1, 1217.1]	1233.8 [1082.4, 1416.5]	1367.8 [1192.9, 1602.4]	1537.3 [1353.5, 1751.2]	1786.2 [1503.9, 2052.2]	1827.8 [1488.1, 2018.1]
**55-59**		891.0 [783.4, 963.0]	952.7 [851.7, 1061.6]	1005.6 [902.4, 1137.4]	1099.3 [985.9, 1239.0]	1200.0 [1050.4, 1355.3]	1370.7 [1218.5, 1556.3]	1538.9 [1335.6, 1720.3]	1647.8 [1481.3, 1778.0]	
**60-64**	860.4 [738.4, 956.1]	872.0 [799.7, 986.9]	937.3 [833.6, 1052.1]	1016.3 [911.6, 1125.3]	1080.4 [971.9, 1227.8]	1206.3 [1071.9, 1344.3]	1347.0 [1175.3, 1577.0]	1407.8 [1242.1, 1619.3]	1592.5 [1488.7, 1777.2]	1640.3 [1418.2, 1877.7]
**65-69**	791.0 [713.8, 879.3]	862.6 [771.8, 980.5]	918.5 [833.4, 1029.6]	980.4 [882.7, 1109.8]	1074.4 [964.0, 1205.4]	1177.7 [1047.0, 1340.0]	1247.7 [1116.6, 1451.8]	1407.5 [1224.7, 1645.0]	1501.6 [1358.7, 1655.3]	
**70-74**	791.4 [681.3, 938.6]	824.8 [734.7, 907.9]	886.8 [798.8, 991.2]	955.0 [860.4, 1063.7]	1019.3 [914.7, 1170.6]	1139.1 [1030.5, 1283.7]	1220.0 [1084.5, 1388.5]	1317.8 [1171.9, 1551.2]	1478.0 [1260.2, 1652.5]	
**75-79**	769.9 [667.6, 858.6]	814.1 [720.7, 909.3]	866.7 [782.8, 965.4]	929.8 [831.3, 1036.3]	996.1 [903.3, 1122.5]	1112.4 [1001.6, 1262.4]	1161.2 [1031.1, 1334.9]	1183.9 [1048.2, 1508.9]		
**80-84**	697.5 [636.9, 766.3]	797.2 [706.3, 903.0]	842.4 [760.8, 947.5]	907.6 [809.3, 1013.6]	989.7 [875.4, 1101.1]	1076.3 [970.6, 1171.7]	1191.4 [1028.0, 1264.0]			
**85-89**	700.1 [619.5, 825.5]	764.1 [696.5, 852.9]	829.2 [720.1, 920.6]	885.5 [797.0, 980.3]	981.9 [839.2, 1078.2]	992.7 [904.9, 1188.8]	957.8 [919.8, 1314.4]			

Pancreas (Male)

Age (years) \ BSA (m^2)	1.3-1.4	1.4-1.5	1.5-1.6	1.6-1.7	1.7-1.8	1.8-1.9	1.9-2.0	2.0-2.1	2.1-2.2	2.2-2.3
**20-24**		55.7<br>[48.3, 65.6]	52.8<br>[45.9, 66.3]	68.3<br>[61.0, 76.7]	70.9<br>[68.7, 80.9]	77.4<br>[72.3, 82.2]	85.4<br>[83.1, 93.0]			
**25-29**			56.9<br>[50.2, 72.4]	65.6<br>[58.5, 76.4]	75.1<br>[66.0, 85.5]	85.7<br>[71.6, 97.1]	86.2<br>[80.2, 98.1]	89.5<br>[77.7, 95.3]		
**30-34**			68.6<br>[57.2, 79.6]	70.1<br>[58.0, 82.7]	74.4<br>[63.9, 84.6]	82.8<br>[68.4, 93.7]	91.5<br>[81.5, 105.5]	96.7<br>[85.3, 103.0]	100.4<br>[77.5, 115.1]	
**35-39**			64.2<br>[47.6, 78.1]	72.5<br>[60.7, 84.9]	79.9<br>[68.2, 90.1]	82.2<br>[73.5, 93.3]	89.8<br>[79.8, 97.1]	92.7<br>[81.3, 106.4]	109.6<br>[98.2, 120.0]	
**40-44**		67.0<br>[49.2, 71.3]	58.8<br>[48.7, 75.3]	68.5<br>[61.9, 79.9]	75.6<br>[64.8, 87.5]	82.4<br>[71.4, 91.5]	87.3<br>[77.9, 97.1]	88.7<br>[72.0, 97.7]	96.3<br>[85.3, 110.3]	
**45-49**		46.8<br>[35.0, 64.8]	63.6<br>[46.9, 76.6]	66.9<br>[57.6, 77.1]	79.5<br>[69.9, 89.2]	80.6<br>[68.3, 92.6]	86.3<br>[75.8, 99.3]	91.9<br>[79.6, 101.6]	103.1<br>[87.9, 112.0]	95.3<br>[79.0, 114.6]
**50-54**		49.4<br>[34.5, 66.2]	58.7<br>[48.2, 70.5]	68.1<br>[56.2, 79.7]	73.6<br>[61.8, 87.4]	79.4<br>[67.5, 90.4]	85.8<br>[73.5, 97.9]	92.3<br>[76.0, 106.4]	100.5<br>[88.5, 112.6]	100.1<br>[87.2, 112.6]
**55-59**		46.2<br>[34.4, 58.1]	57.4<br>[45.9, 65.9]	64.0<br>[51.3, 74.2]	72.0<br>[60.9, 84.2]	78.3<br>[65.1, 90.3]	81.1<br>[67.2, 92.9]	88.2<br>[73.1, 101.5]	92.1<br>[79.2, 101.4]	89.7<br>[82.4, 99.1]
**60-64**	41.4<br>[29.1, 48.2]	43.4<br>[30.2, 53.5]	53.8<br>[41.0, 67.6]	63.5<br>[51.5, 74.8]	70.1<br>[58.7, 82.5]	75.5<br>[63.4, 86.8]	79.3<br>[66.8, 93.0]	82.2<br>[71.7, 96.9]	94.6<br>[77.0, 104.8]	103.7<br>[85.5, 123.6]
**65-69**	33.8<br>[25.9, 45.0]	44.8<br>[33.7, 58.1]	52.8<br>[41.3, 66.3]	59.8<br>[48.8, 71.3]	67.0<br>[55.5, 79.5]	71.7<br>[60.2, 83.9]	77.2<br>[63.3, 89.1]	81.8<br>[66.7, 94.5]	81.2<br>[70.5, 89.8]	89.1<br>[67.0, 104.4]
**70-74**	39.3<br>[25.0, 47.3]	43.8<br>[28.4, 57.5]	52.8<br>[40.6, 65.1]	58.6<br>[46.8, 70.7]	64.9<br>[53.0, 75.9]	69.7<br>[57.9, 81.7]	73.4<br>[61.3, 85.1]	76.3<br>[65.3, 88.8]	75.5<br>[62.3, 95.7]	
**75-79**	31.2<br>[24.0, 42.5]	43.2<br>[32.6, 54.8]	49.1<br>[38.2, 61.0]	54.9<br>[44.7, 66.6]	61.0<br>[49.2, 72.5]	65.1<br>[52.8, 76.4]	69.4<br>[57.6, 79.1]	74.5<br>[62.2, 84.4]	82.5<br>[62.4, 90.4]	
**80-84**	28.4<br>[19.1, 47.0]	41.0<br>[29.4, 52.1]	47.5<br>[35.8, 59.5]	54.1<br>[42.7, 65.2]	57.4<br>[47.0, 69.8]	59.1<br>[50.0, 69.7]	64.1<br>[51.0, 79.2]	72.8<br>[57.6, 88.6]		
**85-89**	36.9<br>[23.3, 50.5]	37.4<br>[26.8, 50.0]	47.6<br>[34.7, 57.7]	48.8<br>[37.9, 58.9]	55.6<br>[45.0, 65.6]	56.8<br>[44.7, 71.2]	69.0<br>[44.4, 77.0]			

Pancreas (Female)

Age (years) \ BSA (m^2)	1.1-1.2	1.2-1.3	1.3-1.4	1.4-1.5	1.5-1.6	1.6-1.7	1.7-1.8	1.8-1.9	1.9-2.0	2.0-2.1
**20-24**		37.7<br>[10.6, 51.5]	48.1<br>[40.6, 59.8]	49.3<br>[43.9, 61.3]	57.7<br>[49.3, 67.1]	62.3<br>[51.8, 72.3]	65.3<br>[51.3, 73.6]			
**25-29**		43.4<br>[29.3, 51.9]	47.8<br>[39.5, 61.3]	55.6<br>[44.1, 61.3]	61.2<br>[52.5, 73.7]	66.8<br>[61.1, 72.6]	70.7<br>[67.3, 82.8]	79.1<br>[66.0, 87.2]		
**30-34**		44.0<br>[22.3, 50.9]	49.0<br>[39.5, 58.0]	54.4<br>[44.5, 62.4]	59.2<br>[49.4, 70.3]	69.0<br>[61.7, 80.0]	72.3<br>[63.8, 86.6]	73.6<br>[65.2, 76.4]	98.0<br>[93.2, 107.4]	
**35-39**		43.0<br>[28.4, 53.6]	48.2<br>[40.4, 54.9]	54.4<br>[47.3, 63.3]	63.0<br>[53.5, 72.5]	66.6<br>[56.1, 74.8]	70.8<br>[62.6, 75.2]	80.2<br>[71.9, 87.6]	76.4<br>[68.7, 83.1]	
**40-44**		34.2<br>[20.1, 44.9]	45.2<br>[33.7, 54.5]	51.5<br>[43.1, 61.4]	60.9<br>[52.4, 70.9]	66.4<br>[58.9, 77.4]	71.4<br>[61.5, 81.7]	83.0<br>[69.1, 91.2]	83.8<br>[71.9, 93.4]	88.1<br>[76.6, 103.4]
**45-49**		33.1<br>[24.5, 40.7]	46.2<br>[34.9, 56.5]	52.8<br>[40.2, 62.7]	59.2<br>[50.5, 69.3]	68.6<br>[57.0, 78.2]	74.7<br>[65.9, 83.7]	77.0<br>[69.1, 88.3]	79.9<br>[65.3, 88.6]	84.1<br>[75.3, 92.8]
**50-54**		40.0<br>[25.6, 48.3]	43.7<br>[30.3, 55.5]	53.1<br>[42.0, 62.3]	60.0<br>[50.7, 69.5]	65.2<br>[55.7, 74.3]	71.5<br>[63.6, 80.6]	76.0<br>[65.5, 88.1]	78.5<br>[69.6, 88.9]	75.0<br>[60.2, 88.2]
**55-59**		38.0<br>[29.5, 45.3]	44.6<br>[33.0, 52.5]	50.6<br>[41.0, 61.4]	59.9<br>[48.9, 69.0]	64.5<br>[53.7, 74.9]	68.8<br>[57.1, 77.6]	75.8<br>[61.7, 84.3]	73.9<br>[66.7, 87.3]	
**60-64**	32.7<br>[16.0, 36.6]	34.2<br>[22.8, 48.2]	44.6<br>[34.8, 54.3]	52.8<br>[42.7, 62.3]	57.6<br>[48.0, 66.6]	63.3<br>[54.4, 74.0]	66.9<br>[56.1, 76.0]	69.0<br>[55.8, 77.8]	74.7<br>[65.4, 81.8]	71.5<br>[61.9, 94.3]
**65-69**	33.3<br>[24.2, 46.0]	36.2<br>[23.2, 47.1]	45.0<br>[34.4, 54.5]	51.4<br>[41.4, 60.3]	56.8<br>[47.9, 65.3]	61.3<br>[51.2, 69.6]	63.9<br>[50.1, 72.3]	65.5<br>[53.2, 77.7]	69.6<br>[62.1, 82.2]	
**70-74**	32.2<br>[23.8, 46.0]	35.5<br>[23.0, 46.4]	41.8<br>[32.7, 51.8]	48.7<br>[39.0, 57.6]	54.2<br>[44.5, 63.7]	57.6<br>[46.9, 70.1]	58.2<br>[50.2, 69.9]	64.2<br>[48.5, 74.5]	58.8<br>[52.1, 69.7]	
**75-79**	30.1<br>[18.7, 42.5]	32.3<br>[21.0, 45.6]	40.1<br>[30.1, 50.1]	46.5<br>[36.9, 55.4]	50.5<br>[41.1, 60.9]	55.9<br>[44.9, 65.1]	56.2<br>[44.2, 63.5]	55.2<br>[47.9, 65.5]		
**80-84**	29.7<br>[19.5, 38.9]	32.1<br>[20.9, 42.3]	39.4<br>[29.5, 49.6]	44.0<br>[35.2, 53.4]	48.2<br>[38.5, 56.6]	51.3<br>[41.9, 60.4]	55.3<br>[42.7, 63.8]			
**85-89**	29.8<br>[19.1, 39.6]	31.9<br>[21.4, 42.3]	36.7<br>[27.6, 46.2]	42.3<br>[32.6, 51.0]	45.4<br>[35.8, 57.2]	46.6<br>[35.6, 53.9]	48.6<br>[29.8, 59.8]			

Spleen (Male)

Age (years) \ BSA (m^2)	1.3-1.4	1.4-1.5	1.5-1.6	1.6-1.7	1.7-1.8	1.8-1.9	1.9-2.0	2.0-2.1	2.1-2.2	2.2-2.3
**20-24**		178.8<br>[103.3, 234.3]	161.1<br>[104.3, 210.8]	165.1<br>[142.2, 257.5]	181.8<br>[149.6, 220.2]	198.3<br>[146.8, 227.8]	247.9<br>[220.5, 264.2]			
**25-29**			125.3<br>[96.2, 179.8]	145.4<br>[118.4, 221.0]	161.7<br>[112.6, 201.8]	193.3<br>[154.0, 253.9]	164.7<br>[145.4, 279.0]	241.7<br>[195.0, 291.3]		
**30-34**			155.5<br>[111.0, 190.0]	159.7<br>[129.7, 219.8]	151.6<br>[120.9, 199.3]	166.8<br>[129.2, 209.7]	197.3<br>[146.8, 232.3]	212.8<br>[159.9, 278.9]	206.7<br>[167.2, 276.1]	
**35-39**			134.7<br>[97.6, 193.3]	139.6<br>[104.8, 241.7]	153.1<br>[105.8, 221.2]	166.9<br>[122.7, 209.5]	182.7<br>[142.7, 222.4]	192.7<br>[160.6, 232.5]	285.5<br>[193.1, 407.0]	
**40-44**		137.1<br>[93.8, 205.3]	118.7<br>[83.7, 201.2]	131.4<br>[87.6, 177.3]	144.9<br>[108.0, 192.2]	151.6<br>[117.0, 202.5]	177.0<br>[126.1, 228.5]	198.5<br>[148.2, 263.8]	180.1<br>[151.2, 236.5]	
**45-49**		92.0<br>[67.7, 148.3]	98.8<br>[70.0, 139.6]	114.3<br>[87.0, 167.0]	132.1<br>[100.8, 188.1]	149.1<br>[115.0, 197.4]	150.4<br>[119.1, 193.1]	168.5<br>[132.4, 228.0]	236.2<br>[183.2, 330.4]	239.4<br>[169.2, 375.0]
**50-54**		90.8<br>[66.5, 151.6]	102.5<br>[71.7, 140.4]	107.9<br>[79.4, 152.6]	124.4<br>[91.0, 168.6]	147.2<br>[105.5, 182.5]	154.6<br>[118.2, 207.4]	192.2<br>[144.3, 244.5]	208.1<br>[160.0, 251.8]	210.5<br>[190.1, 277.1]
**55-59**		94.4<br>[65.4, 137.6]	91.6<br>[59.9, 139.1]	105.1<br>[76.4, 147.1]	120.5<br>[90.5, 163.0]	132.5<br>[96.4, 175.6]	147.6<br>[112.6, 197.9]	171.2<br>[124.1, 232.1]	169.1<br>[121.4, 252.0]	233.1<br>[173.3, 274.7]
**60-64**	90.9<br>[73.0, 133.0]	92.2<br>[53.3, 114.2]	90.4<br>[63.6, 133.0]	97.8<br>[70.7, 135.7]	117.0<br>[88.2, 164.3]	136.0<br>[101.4, 179.5]	149.6<br>[113.0, 204.2]	163.0<br>[123.7, 226.7]	189.0<br>[141.6, 229.0]	200.3<br>[145.0, 252.1]
**65-69**	77.7<br>[48.8, 119.5]	85.5<br>[53.8, 112.8]	90.7<br>[63.0, 144.9]	102.3<br>[76.4, 145.1]	122.0<br>[87.3, 170.1]	133.2<br>[98.3, 183.6]	145.6<br>[106.8, 200.1]	159.0<br>[116.0, 223.3]	170.7<br>[108.0, 215.1]	214.3<br>[193.2, 252.9]
**70-74**	73.5<br>[49.6, 103.7]	84.3<br>[57.1, 122.1]	92.1<br>[67.5, 129.2]	104.1<br>[72.7, 139.7]	116.6<br>[84.9, 159.3]	131.1<br>[95.2, 181.3]	146.4<br>[106.3, 194.7]	171.4<br>[127.2, 228.8]	187.3<br>[148.1, 296.1]	
**75-79**	80.0<br>[48.8, 108.6]	86.6<br>[58.9, 125.2]	93.2<br>[68.9, 131.1]	108.0<br>[78.7, 146.9]	116.8<br>[85.2, 159.7]	126.3<br>[95.1, 172.6]	125.9<br>[94.9, 168.1]	149.3<br>[118.6, 215.4]	187.4<br>[146.4, 221.1]	
**80-84**	67.4<br>[49.4, 97.6]	83.0<br>[56.1, 114.5]	93.2<br>[67.3, 131.8]	102.4<br>[74.8, 141.9]	114.5<br>[84.5, 151.0]	115.9<br>[90.7, 155.9]	142.0<br>[106.2, 187.8]	169.5<br>[120.9, 218.0]		
**85-89**	60.8<br>[48.2, 90.8]	80.8<br>[55.9, 117.2]	90.4<br>[63.0, 122.6]	95.9<br>[70.3, 133.8]	107.0<br>[77.7, 136.3]	119.5<br>[93.0, 164.7]	133.4<br>[95.8, 177.7]			

Spleen (Female)

Age (years) \ BSA (m^2)	1.1-1.2	1.2-1.3	1.3-1.4	1.4-1.5	1.5-1.6	1.6-1.7	1.7-1.8	1.8-1.9	1.9-2.0	2.0-2.1
**20-24**		147.4<br>[94.3, 185.8]	121.7<br>[93.1, 175.9]	133.7<br>[100.9, 164.9]	144.9<br>[120.0, 185.6]	174.1<br>[136.3, 184.6]	147.9<br>[142.2, 191.0]			
**25-29**		123.1<br>[77.5, 167.2]	117.0<br>[90.0, 142.0]	126.2<br>[94.4, 161.9]	147.6<br>[115.4, 186.1]	151.1<br>[124.6, 185.1]	191.1<br>[144.2, 283.3]	197.7<br>[170.0, 216.1]		
**30-34**		90.8<br>[66.5, 116.9]	120.2<br>[95.2, 170.4]	126.3<br>[94.1, 167.3]	136.9<br>[115.0, 192.6]	147.2<br>[113.5, 200.4]	161.9<br>[137.1, 195.4]	156.1<br>[139.1, 226.0]	246.7<br>[219.6, 285.3]	
**35-39**		77.2<br>[56.0, 99.7]	99.1<br>[80.7, 129.8]	117.2<br>[94.3, 144.8]	131.4<br>[101.7, 171.6]	140.7<br>[109.7, 186.4]	153.6<br>[121.1, 181.7]	184.3<br>[145.6, 222.8]	206.6<br>[171.2, 264.2]	
**40-44**		76.7<br>[69.2, 104.2]	100.0<br>[74.3, 140.4]	104.0<br>[83.5, 143.6]	117.5<br>[91.7, 150.2]	136.3<br>[106.5, 173.4]	153.8<br>[123.3, 189.0]	172.3<br>[134.3, 221.9]	155.9<br>[125.6, 221.0]	209.9<br>[178.8, 309.0]
**45-49**		84.5<br>[59.7, 143.2]	93.3<br>[72.4, 127.4]	100.4<br>[76.7, 130.4]	109.2<br>[89.2, 143.1]	124.6<br>[96.8, 167.6]	135.0<br>[106.5, 172.4]	164.4<br>[123.0, 181.6]	195.6<br>[140.5, 230.4]	199.0<br>[186.0, 273.7]
**50-54**		82.6<br>[60.0, 122.4]	80.5<br>[57.7, 112.8]	94.3<br>[73.0, 128.1]	108.8<br>[86.8, 137.6]	121.2<br>[95.1, 156.4]	136.9<br>[102.4, 190.5]	172.2<br>[132.6, 229.7]	193.6<br>[157.0, 230.1]	159.4<br>[152.4, 221.1]
**55-59**		70.5<br>[48.3, 103.0]	80.9<br>[60.5, 114.8]	91.4<br>[67.7, 121.0]	103.0<br>[75.8, 140.5]	109.7<br>[84.8, 144.4]	119.3<br>[87.9, 163.3]	164.5<br>[122.9, 207.6]	172.9<br>[126.9, 205.5]	
**60-64**	84.1<br>[51.5, 127.8]	82.7<br>[55.7, 121.7]	82.2<br>[54.1, 104.0]	85.3<br>[62.1, 120.4]	98.1<br>[72.3, 132.0]	109.4<br>[80.9, 152.0]	129.2<br>[94.3, 166.8]	140.1<br>[105.0, 179.2]	179.0<br>[146.6, 217.5]	202.0<br>[146.1, 252.6]
**65-69**	80.9<br>[36.1, 117.1]	75.6<br>[56.8, 103.8]	80.2<br>[60.8, 109.9]	85.6<br>[64.0, 117.8]	94.5<br>[71.3, 124.7]	108.0<br>[80.1, 144.2]	122.5<br>[83.9, 170.1]	138.5<br>[108.5, 176.6]	142.3<br>[112.9, 166.7]	
**70-74**	68.0<br>[51.5, 99.0]	64.2<br>[47.3, 93.8]	74.3<br>[53.8, 101.6]	81.7<br>[61.6, 109.9]	93.8<br>[69.7, 126.0]	108.6<br>[81.3, 142.5]	114.7<br>[86.0, 153.7]	130.1<br>[97.1, 159.3]	135.7<br>[110.3, 182.2]	
**75-79**	62.0<br>[34.4, 93.0]	64.5<br>[45.1, 92.5]	72.6<br>[52.7, 100.3]	80.7<br>[60.1, 109.8]	89.6<br>[66.2, 118.5]	99.7<br>[74.8, 139.0]	110.9<br>[83.0, 136.4]	155.0<br>[117.6, 203.9]		
**80-84**	56.7<br>[41.6, 68.9]	64.5<br>[48.0, 95.4]	69.7<br>[51.6, 98.2]	77.0<br>[57.2, 106.1]	85.9<br>[64.5, 118.3]	85.9<br>[62.2, 123.1]	113.8<br>[89.8, 143.5]			
**85-89**	57.8<br>[38.2, 84.1]	58.4<br>[44.1, 78.7]	71.7<br>[52.1, 97.3]	74.9<br>[55.1, 99.8]	84.4<br>[62.6, 110.9]	91.2<br>[69.1, 123.7]	118.6<br>[90.8, 143.7]			

Kidney right (Male)

Age (years) \ BSA (m^2)	1.3-1.4	1.4-1.5	1.5-1.6	1.6-1.7	1.7-1.8	1.8-1.9	1.9-2.0	2.0-2.1	2.1-2.2	2.2-2.3
**20-24**		101.9 [89.0, 133.1]	110.8 [98.4, 138.0]	121.8 [111.2, 143.3]	131.6 [119.4, 143.8]	148.0 [123.9, 161.5]	149.6 [142.7, 170.8]			
**25-29**			103.6 [94.9, 120.6]	121.6 [110.3, 137.0]	136.1 [120.4, 147.7]	156.9 [132.4, 171.2]	161.0 [140.2, 172.9]	160.2 [152.5, 188.8]		
**30-34**			123.8 [99.0, 140.1]	130.8 [115.1, 143.8]	137.3 [121.9, 155.6]	152.2 [141.5, 163.9]	159.4 [144.3, 188.2]	164.4 [144.4, 186.2]	198.9 [178.8, 211.7]	
**35-39**			109.6 [95.1, 124.8]	133.3 [115.4, 157.8]	141.5 [120.1, 163.5]	150.2 [136.1, 175.5]	156.7 [138.5, 181.8]	175.5 [144.9, 193.0]	202.9 [179.4, 240.8]	
**40-44**		117.0 [102.3, 129.7]	114.1 [95.1, 134.3]	123.8 [109.3, 145.3]	136.8 [121.9, 159.2]	157.9 [138.4, 181.4]	161.7 [143.6, 187.4]	188.1 [169.4, 210.0]	176.9 [163.6, 191.1]	
**45-49**		77.0 [64.0, 109.6]	120.1 [102.8, 142.2]	124.5 [110.1, 145.1]	142.1 [128.2, 163.9]	149.7 [131.1, 173.7]	166.8 [146.7, 188.2]	191.0 [166.2, 215.5]	200.6 [166.9, 240.8]	195.8 [169.7, 225.7]
**50-54**		112.1 [98.2, 119.2]	119.7 [104.2, 136.2]	132.4 [115.3, 149.8]	145.7 [123.0, 165.8]	155.4 [138.3, 176.6]	166.6 [144.9, 193.7]	189.1 [165.8, 220.4]	188.6 [167.1, 224.4]	201.2 [167.1, 233.8]
**55-59**		104.4 [91.4, 120.9]	119.3 [106.0, 138.2]	128.9 [109.9, 146.0]	144.1 [124.5, 164.5]	153.9 [133.9, 175.7]	168.6 [148.6, 191.4]	178.8 [158.1, 197.6]	186.1 [159.1, 222.2]	210.6 [172.1, 238.4]
**60-64**	96.1 [83.7, 103.2]	102.7 [87.8, 113.6]	117.5 [99.1, 134.0]	132.0 [114.9, 148.5]	144.5 [122.7, 163.3]	152.2 [131.9, 171.3]	163.2 [141.6, 188.5]	172.2 [154.9, 196.2]	192.8 [172.4, 214.8]	212.1 [197.1, 231.5]
**65-69**	93.8 [76.2, 110.1]	107.6 [91.4, 126.3]	113.2 [96.2, 132.6]	128.2 [110.6, 146.5]	139.8 [121.4, 158.2]	152.0 [131.9, 171.5]	165.6 [142.1, 187.3]	174.9 [150.7, 195.4]	194.8 [166.0, 214.2]	193.6 [170.6, 223.6]
**70-74**	91.2 [76.8, 103.2]	102.2 [82.6, 118.4]	116.8 [100.3, 134.9]	127.1 [109.2, 145.2]	138.2 [119.3, 157.2]	148.3 [127.9, 169.0]	158.3 [137.7, 182.1]	167.0 [146.5, 189.9]	187.8 [147.2, 242.3]	
**75-79**	94.8 [81.1, 110.9]	100.8 [84.4, 116.5]	111.5 [95.3, 129.3]	123.6 [105.7, 143.6]	132.6 [115.1, 151.9]	144.0 [124.4, 166.8]	150.7 [133.9, 175.1]	162.6 [144.1, 182.0]	181.2 [165.5, 204.3]	
**80-84**	86.4 [70.4, 110.1]	95.8 [79.2, 114.5]	108.8 [94.3, 124.5]	119.4 [102.3, 138.6]	130.2 [112.1, 148.8]	135.9 [118.3, 153.8]	142.6 [122.0, 164.8]	157.9 [132.0, 188.3]		
**85-89**	84.9 [66.0, 95.8]	95.1 [79.0, 108.6]	104.1 [85.9, 120.3]	114.0 [99.5, 132.0]	125.8 [110.8, 143.6]	128.4 [112.4, 154.6]	147.8 [125.4, 169.7]			

Kidney right (Female)

Age (years) \ BSA (m^2)	1.1-1.2	1.2-1.3	1.3-1.4	1.4-1.5	1.5-1.6	1.6-1.7	1.7-1.8	1.8-1.9	1.9-2.0	2.0-2.1
**20-24**		81.9 [60.3, 103.9]	97.8 [88.3, 108.8]	98.3 [90.3, 117.3]	109.4 [97.8, 125.3]	132.6 [118.7, 138.1]	145.3 [127.4, 165.8]			
**25-29**		83.3 [55.7, 97.0]	95.6 [83.2, 110.8]	107.0 [93.6, 121.0]	114.2 [101.0, 134.3]	131.7 [113.6, 145.4]	139.1 [129.7, 168.7]	144.8 [134.0, 164.1]		
**30-34**		71.8 [64.8, 83.2]	100.4 [85.2, 111.8]	109.1 [99.3, 121.8]	116.3 [100.4, 132.3]	129.9 [114.1, 153.3]	140.0 [126.4, 148.8]	164.9 [134.7, 180.3]	181.3 [163.0, 196.5]	
**35-39**		95.6 [81.1, 118.4]	96.1 [85.8, 113.1]	107.6 [93.8, 121.7]	117.5 [105.8, 133.9]	125.8 [114.2, 147.3]	141.8 [123.4, 162.0]	154.8 [136.9, 167.9]	157.7 [149.8, 183.8]	
**40-44**		86.9 [72.5, 94.3]	98.2 [83.8, 111.1]	104.3 [92.3, 117.5]	121.7 [107.8, 134.1]	130.2 [113.6, 145.1]	142.4 [118.1, 159.6]	159.8 [142.8, 184.3]	167.8 [155.0, 185.6]	210.5 [193.6, 225.5]
**45-49**		80.3 [70.9, 91.8]	96.4 [84.0, 112.1]	106.6 [93.0, 118.1]	116.4 [103.1, 131.4]	128.4 [113.7, 144.9]	136.4 [123.3, 153.4]	152.1 [133.3, 173.4]	165.1 [152.7, 183.2]	157.4 [132.7, 200.2]
**50-54**		83.6 [67.5, 96.1]	95.8 [83.1, 109.5]	104.7 [91.4, 120.4]	117.4 [103.1, 131.7]	126.7 [111.8, 144.3]	141.9 [120.4, 161.0]	154.7 [135.5, 180.3]	168.4 [141.6, 196.9]	165.3 [143.8, 197.7]
**55-59**		86.6 [69.1, 96.1]	95.7 [81.4, 112.5]	104.7 [91.5, 119.3]	116.1 [100.9, 130.0]	124.3 [109.6, 139.5]	136.2 [116.7, 156.9]	146.1 [130.4, 165.4]	164.2 [141.5, 181.1]	
**60-64**	70.0 [61.0, 83.5]	86.7 [68.0, 96.8]	96.1 [82.4, 111.1]	104.9 [91.2, 120.0]	115.0 [99.7, 130.5]	124.2 [109.7, 144.1]	133.2 [118.9, 151.0]	136.7 [120.2, 155.6]	160.0 [143.4, 184.5]	171.5 [142.0, 183.9]
**65-69**	73.5 [58.5, 95.3]	86.9 [72.6, 99.7]	95.8 [83.2, 111.0]	104.8 [90.0, 116.8]	113.8 [100.5, 129.3]	125.1 [109.0, 143.0]	133.0 [116.8, 150.8]	148.4 [125.4, 172.8]	153.0 [140.3, 163.5]	
**70-74**	70.8 [57.5, 92.9]	86.5 [68.7, 99.2]	95.6 [81.6, 109.2]	104.1 [90.7, 120.3]	114.4 [97.8, 130.6]	126.2 [105.0, 139.9]	133.5 [115.4, 155.0]	143.1 [123.4, 168.8]	145.5 [127.6, 187.9]	
**75-79**	68.8 [53.9, 78.0]	82.2 [70.7, 96.5]	94.5 [82.1, 109.1]	102.2 [88.0, 117.9]	112.0 [97.5, 127.4]	122.1 [105.8, 137.1]	127.5 [107.7, 138.7]	133.5 [117.9, 151.8]		
**80-84**	79.2 [67.0, 90.0]	81.8 [67.1, 94.1]	92.3 [77.5, 107.3]	102.1 [87.8, 117.8]	108.6 [94.4, 127.4]	114.0 [99.6, 127.6]	126.8 [106.2, 147.2]			
**85-89**	73.3 [59.6, 88.0]	79.0 [66.8, 94.1]	89.3 [76.6, 102.9]	95.8 [82.3, 110.0]	102.2 [89.5, 119.3]	110.8 [92.9, 132.6]	117.6 [82.7, 126.1]			

Kidney left (Male)

Age (years) \ BSA (m^2)	1.3-1.4	1.4-1.5	1.5-1.6	1.6-1.7	1.7-1.8	1.8-1.9	1.9-2.0	2.0-2.1	2.1-2.2	2.2-2.3
**20-24**		112.9 [98.4, 126.9]	115.0 [105.1, 140.4]	133.0 [122.5, 157.4]	139.1 [130.9, 151.5]	159.6 [137.4, 171.9]	154.9 [146.2, 185.3]			
**25-29**			106.8 [68.6, 132.6]	128.2 [107.5, 139.6]	145.8 [129.2, 160.7]	155.6 [141.4, 191.8]	171.0 [149.5, 183.5]	166.4 [150.0, 198.1]		
**30-34**			122.3 [110.7, 141.2]	135.5 [119.6, 153.5]	142.2 [127.5, 163.4]	160.8 [145.5, 171.9]	166.9 [145.8, 181.1]	161.1 [154.4, 168.4]	209.8 [174.7, 217.2]	
**35-39**			125.7 [87.3, 136.1]	136.8 [118.5, 157.5]	149.4 [132.2, 168.1]	159.2 [140.0, 178.7]	162.1 [142.5, 183.9]	177.2 [154.6, 199.5]	194.1 [162.7, 222.8]	
**40-44**		125.8 [81.2, 139.4]	126.8 [98.9, 140.7]	136.3 [110.9, 149.9]	140.4 [125.0, 155.2]	160.4 [138.9, 181.3]	172.0 [152.2, 194.7]	192.4 [148.5, 209.3]	188.4 [158.0, 209.6]	
**45-49**		104.4 [77.7, 120.9]	122.1 [106.0, 146.9]	131.7 [108.1, 151.7]	150.5 [130.7, 174.3]	157.9 [138.1, 179.3]	173.4 [156.4, 198.9]	189.9 [166.5, 208.6]	196.5 [165.6, 226.9]	203.5 [188.5, 248.0]
**50-54**		112.4 [90.3, 130.1]	128.1 [114.1, 143.6]	137.3 [118.2, 155.1]	150.5 [126.9, 171.9]	164.4 [141.3, 186.8]	171.8 [151.5, 198.6]	190.6 [168.7, 213.1]	199.2 [178.2, 233.8]	213.4 [185.1, 246.6]
**55-59**		115.8 [90.1, 133.7]	130.8 [108.9, 146.1]	137.4 [119.4, 157.3]	149.3 [131.3, 172.8]	159.7 [139.4, 184.2]	172.4 [150.6, 197.2]	183.8 [163.2, 205.7]	192.8 [162.5, 222.3]	209.0 [169.5, 235.5]
**60-64**	86.4 [80.5, 112.0]	109.5 [98.3, 127.7]	123.8 [106.9, 139.1]	137.2 [121.0, 153.6]	146.7 [126.3, 169.0]	156.3 [136.4, 177.8]	166.8 [146.3, 190.5]	175.4 [153.0, 198.8]	198.3 [171.6, 220.3]	215.0 [188.5, 225.3]
**65-69**	93.5 [72.5, 111.4]	113.6 [97.1, 132.3]	119.1 [102.2, 137.7]	133.5 [115.7, 152.8]	142.7 [122.1, 163.4]	154.1 [134.0, 174.8]	166.1 [142.8, 189.7]	174.1 [149.2, 196.3]	189.5 [166.9, 214.0]	189.5 [180.1, 215.0]
**70-74**	96.4 [79.1, 113.7]	110.5 [89.3, 129.0]	122.1 [103.8, 139.5]	131.8 [112.5, 151.6]	142.6 [122.5, 162.6]	152.9 [132.1, 175.9]	161.5 [138.8, 185.7]	169.7 [147.4, 194.4]	181.3 [145.2, 224.4]	
**75-79**	96.8 [83.0, 118.7]	106.5 [88.2, 125.9]	117.0 [96.9, 134.9]	128.5 [110.5, 148.1]	136.7 [118.4, 158.6]	149.4 [127.7, 171.6]	156.5 [134.7, 179.1]	170.4 [144.2, 192.2]	171.8 [152.1, 195.5]	
**80-84**	88.4 [71.5, 106.9]	100.8 [83.0, 123.0]	114.3 [96.1, 132.0]	122.4 [103.3, 141.3]	134.3 [116.0, 155.2]	139.9 [117.5, 159.8]	146.3 [124.7, 174.4]	158.2 [147.5, 177.8]		
**85-89**	84.2 [68.3, 105.3]	94.8 [82.1, 111.5]	106.8 [89.4, 128.5]	119.1 [100.5, 140.9]	130.5 [109.8, 154.1]	132.8 [111.6, 151.5]	149.0 [126.3, 172.3]			

Kidney left (Female)

Age (years) \ BSA (m^2)	1.1-1.2	1.2-1.3	1.3-1.4	1.4-1.5	1.5-1.6	1.6-1.7	1.7-1.8	1.8-1.9	1.9-2.0	2.0-2.1
**20-24**		69.3 [46.3, 103.3]	107.4 [94.4, 117.5]	106.0 [86.0, 132.0]	118.2 [104.5, 136.4]	133.4 [121.4, 145.5]	145.5 [127.0, 163.8]			
**25-29**		84.2 [73.5, 108.1]	107.1 [95.8, 115.0]	112.5 [99.3, 126.7]	124.1 [115.6, 141.8]	137.7 [126.8, 151.7]	157.0 [137.4, 176.3]	156.2 [139.8, 169.5]		
**30-34**			107.8 [84.2, 124.0]	113.9 [104.6, 130.4]	127.9 [110.6, 145.6]	138.4 [124.8, 167.9]	149.4 [131.0, 165.1]	150.6 [142.3, 198.2]	191.5 [185.7, 200.7]	
**35-39**		98.2 [80.9, 131.2]	102.2 [87.3, 117.4]	112.7 [98.9, 125.8]	123.8 [107.3, 140.7]	135.1 [121.7, 156.2]	154.2 [128.6, 176.5]	161.9 [140.9, 174.2]	169.6 [154.1, 192.4]	
**40-44**		86.5 [70.6, 101.2]	100.7 [87.5, 115.0]	110.8 [96.4, 127.0]	125.9 [112.5, 142.2]	134.5 [119.7, 155.4]	150.6 [126.2, 170.1]	164.8 [142.8, 183.2]	172.6 [162.6, 192.6]	211.5 [200.9, 256.2]
**45-49**		85.2 [55.1, 98.2]	103.6 [88.4, 118.0]	111.2 [99.3, 127.4]	124.3 [111.0, 141.5]	135.1 [119.3, 151.5]	146.3 [129.2, 160.4]	164.6 [146.4, 178.0]	167.4 [151.2, 178.2]	179.5 [143.0, 217.9]
**50-54**		86.0 [63.5, 107.2]	102.2 [87.1, 114.9]	113.8 [98.1, 128.0]	124.0 [109.1, 139.8]	133.4 [117.7, 151.5]	150.0 [127.9, 169.5]	157.5 [138.1, 178.5]	169.7 [150.4, 198.3]	183.1 [143.5, 212.3]
**55-59**		85.1 [62.1, 106.8]	103.5 [87.5, 116.2]	109.9 [96.9, 125.6]	120.9 [104.8, 135.1]	130.9 [112.7, 146.3]	145.4 [123.2, 162.3]	153.0 [131.0, 165.2]	167.8 [149.3, 189.7]	
**60-64**	74.9 [35.4, 84.8]	92.4 [73.2, 106.0]	99.2 [83.5, 116.6]	113.2 [98.6, 128.7]	121.9 [105.3, 138.3]	130.3 [115.3, 147.9]	140.0 [118.3, 156.7]	143.3 [121.9, 165.4]	163.1 [149.4, 193.4]	177.3 [155.6, 252.4]
**65-69**	84.5 [73.2, 100.6]	93.8 [73.3, 106.9]	101.2 [86.7, 116.9]	111.5 [96.7, 126.4]	120.8 [105.9, 136.7]	130.1 [112.9, 148.5]	136.2 [117.9, 151.4]	143.3 [124.1, 173.9]	146.3 [139.8, 163.9]	
**70-74**	74.7 [47.9, 86.9]	91.7 [74.9, 110.1]	101.0 [85.1, 115.9]	109.1 [93.7, 128.2]	119.3 [103.8, 136.5]	128.0 [108.6, 148.4]	136.0 [114.2, 160.2]	154.3 [124.3, 177.3]	129.9 [122.0, 139.8]	
**75-79**	77.3 [62.0, 87.7]	90.3 [75.3, 106.5]	99.8 [85.0, 114.3]	109.3 [92.3, 126.4]	117.0 [101.2, 133.1]	124.6 [106.7, 144.1]	127.2 [106.9, 148.7]	125.1 [97.6, 144.5]		
**80-84**	79.8 [67.6, 94.5]	86.1 [69.0, 101.4]	97.0 [82.2, 113.4]	106.1 [90.6, 124.3]	113.7 [97.8, 132.9]	117.6 [104.4, 132.9]	130.4 [111.8, 146.0]			
**85-89**	74.6 [59.0, 96.1]	81.6 [68.4, 97.0]	94.4 [80.0, 109.0]	100.3 [83.7, 113.9]	108.9 [89.2, 127.3]	114.4 [92.4, 135.3]	125.0 [93.8, 134.3]			

Kidney total (Male)

Age (years) \ BSA (m^2)	1.3-1.4	1.4-1.5	1.5-1.6	1.6-1.7	1.7-1.8	1.8-1.9	1.9-2.0	2.0-2.1	2.1-2.2	2.2-2.3
**20-24**		213.6<br>[190.8, 262.4]	234.6<br>[200.5, 272.3]	253.1<br>[229.8, 290.9]	268.2<br>[257.3, 293.5]	309.2<br>[268.2, 334.6]	304.5<br>[288.9, 356.1]			
**25-29**			207.2<br>[166.6, 252.9]	252.8<br>[209.5, 277.0]	279.4<br>[258.4, 303.8]	316.8<br>[276.5, 360.8]	317.4<br>[289.4, 345.1]	321.7<br>[296.4, 385.3]		
**30-34**			247.2<br>[210.7, 281.9]	265.7<br>[228.4, 289.5]	278.7<br>[246.6, 307.3]	311.7<br>[286.6, 332.9]	327.5<br>[292.1, 368.0]	324.6<br>[304.1, 363.6]	407.5<br>[344.6, 424.5]	
**35-39**			234.7<br>[192.1, 258.9]	271.6<br>[235.3, 305.5]	286.7<br>[254.8, 329.5]	303.7<br>[280.3, 358.7]	315.6<br>[281.1, 354.2]	337.0<br>[294.8, 385.9]	385.6<br>[344.5, 438.8]	
**40-44**		242.8<br>[183.4, 270.1]	246.2<br>[197.9, 277.1]	262.2<br>[220.1, 295.8]	276.8<br>[252.0, 310.0]	315.1<br>[276.3, 355.7]	333.0<br>[300.4, 383.7]	369.5<br>[321.3, 410.5]	367.3<br>[325.0, 394.5]	
**45-49**		186.3<br>[129.4, 221.7]	234.5<br>[199.2, 281.3]	255.7<br>[207.7, 294.6]	291.0<br>[261.4, 329.7]	306.9<br>[268.3, 346.1]	331.9<br>[286.2, 379.7]	369.3<br>[328.3, 401.6]	400.7<br>[351.4, 476.4]	396.3<br>[360.6, 469.0]
**50-54**		222.7<br>[175.6, 245.1]	243.5<br>[214.5, 267.6]	265.1<br>[234.7, 302.6]	291.8<br>[248.5, 331.2]	312.7<br>[272.0, 351.2]	332.7<br>[290.1, 375.2]	374.1<br>[327.4, 419.4]	387.4<br>[341.4, 436.4]	400.3<br>[345.0, 446.7]
**55-59**		214.0<br>[180.9, 249.7]	248.3<br>[212.9, 285.6]	264.0<br>[228.3, 298.6]	289.8<br>[253.5, 332.0]	308.7<br>[268.0, 350.7]	338.1<br>[293.2, 375.4]	357.5<br>[320.3, 395.4]	367.9<br>[325.7, 428.0]	407.6<br>[351.2, 437.4]
**60-64**	181.5<br>[145.9, 212.6]	211.7<br>[191.0, 236.9]	237.4<br>[210.6, 271.7]	265.3<br>[232.7, 299.5]	285.3<br>[245.1, 321.3]	305.6<br>[265.0, 345.1]	328.7<br>[287.6, 370.5]	344.7<br>[300.4, 383.6]	371.1<br>[337.7, 414.4]	431.6<br>[391.4, 448.1]
**65-69**	183.9<br>[156.1, 217.5]	215.1<br>[188.1, 249.0]	230.8<br>[195.0, 263.8]	255.7<br>[221.5, 290.5]	278.3<br>[239.7, 317.0]	300.6<br>[261.9, 339.2]	321.4<br>[277.9, 368.9]	345.7<br>[296.8, 391.0]	365.5<br>[331.2, 444.9]	376.5<br>[350.3, 430.9]
**70-74**	182.8<br>[152.0, 206.2]	208.7<br>[172.9, 241.1]	237.3<br>[200.9, 271.2]	254.7<br>[220.7, 291.5]	273.9<br>[236.2, 312.0]	295.1<br>[255.2, 337.4]	315.2<br>[273.4, 358.5]	332.5<br>[291.5, 379.7]	343.8<br>[295.1, 444.9]	
**75-79**	187.7<br>[169.8, 224.6]	203.5<br>[170.0, 234.4]	225.6<br>[190.3, 258.6]	246.8<br>[212.4, 284.0]	264.9<br>[230.5, 302.9]	287.0<br>[248.8, 330.9]	303.5<br>[261.7, 342.5]	328.8<br>[276.0, 370.5]	346.1<br>[318.8, 396.0]	
**80-84**	170.0<br>[146.0, 206.6]	194.9<br>[165.2, 226.5]	220.6<br>[186.2, 252.2]	235.9<br>[203.0, 272.7]	258.9<br>[222.1, 294.7]	268.6<br>[229.2, 309.4]	276.6<br>[242.6, 334.1]	309.4<br>[269.5, 370.8]		
**85-89**	159.8<br>[124.5, 196.7]	185.9<br>[159.5, 216.8]	208.7<br>[174.7, 242.8]	229.7<br>[194.1, 267.4]	253.0<br>[215.9, 290.8]	253.7<br>[215.6, 287.6]	294.4<br>[241.8, 331.2]			

Kidney total (Female)

Age (years) \ BSA (m^2)	1.1-1.2	1.2-1.3	1.3-1.4	1.4-1.5	1.5-1.6	1.6-1.7	1.7-1.8	1.8-1.9	1.9-2.0	2.0-2.1
**20-24**		167.9<br>[131.0, 208.1]	204.9<br>[179.5, 220.1]	202.9<br>[181.2, 240.7]	222.3<br>[208.4, 259.3]	264.9<br>[245.1, 285.8]	285.6<br>[253.5, 320.6]			
**25-29**		166.3<br>[122.3, 205.1]	200.0<br>[180.4, 223.8]	218.8<br>[193.4, 243.7]	238.2<br>[218.5, 272.4]	270.5<br>[242.8, 298.5]	315.3<br>[268.2, 327.7]	303.9<br>[273.7, 320.3]		
**30-34**		150.0<br>[93.9, 171.3]	207.6<br>[178.5, 235.6]	224.0<br>[202.6, 245.4]	243.7<br>[209.9, 274.7]	269.1<br>[243.6, 315.2]	286.9<br>[258.1, 312.1]	309.2<br>[275.6, 379.3]	372.9<br>[348.7, 397.2]	
**35-39**		192.8<br>[159.3, 249.2]	196.2<br>[171.2, 226.9]	219.4<br>[190.3, 242.8]	240.2<br>[213.7, 270.2]	259.9<br>[233.8, 300.7]	300.5<br>[248.9, 337.6]	318.3<br>[280.9, 340.3]	322.7<br>[307.3, 375.2]	
**40-44**		163.1<br>[154.3, 185.1]	197.7<br>[175.1, 224.9]	214.7<br>[185.8, 238.1]	244.2<br>[221.2, 276.3]	260.9<br>[232.2, 298.8]	283.0<br>[240.5, 327.7]	329.1<br>[285.7, 359.9]	341.0<br>[317.7, 384.3]	422.2<br>[391.7, 494.2]
**45-49**		157.4<br>[119.8, 185.3]	200.7<br>[169.8, 224.3]	213.6<br>[194.2, 244.3]	240.2<br>[214.1, 267.2]	260.0<br>[233.8, 294.4]	273.9<br>[252.0, 313.0]	311.4<br>[281.1, 341.3]	331.8<br>[304.6, 354.8]	337.0<br>[278.7, 425.4]
**50-54**		162.5<br>[132.8, 205.0]	195.7<br>[169.9, 221.5]	216.4<br>[188.8, 242.1]	238.4<br>[212.4, 267.6]	257.8<br>[229.5, 287.4]	290.7<br>[252.3, 323.5]	310.0<br>[268.9, 352.4]	336.2<br>[295.2, 394.5]	356.4<br>[297.4, 409.3]
**55-59**		168.5<br>[132.7, 203.7]	198.4<br>[165.9, 224.4]	211.9<br>[188.6, 241.0]	236.2<br>[206.3, 261.2]	253.0<br>[221.7, 283.0]	277.5<br>[239.3, 314.2]	300.5<br>[267.6, 328.6]	336.1<br>[299.3, 355.1]	
**60-64**	144.6<br>[83.2, 161.1]	170.9<br>[134.5, 200.5]	195.2<br>[168.5, 224.7]	215.0<br>[186.7, 244.2]	233.3<br>[203.5, 262.2]	250.7<br>[222.5, 285.5]	272.2<br>[238.4, 307.2]	280.9<br>[240.2, 317.8]	308.8<br>[282.7, 371.8]	342.9<br>[259.9, 366.8]
**65-69**	168.9<br>[128.2, 181.9]	177.5<br>[150.8, 201.7]	193.8<br>[168.0, 220.6]	214.8<br>[186.9, 240.0]	232.5<br>[204.8, 260.8]	253.4<br>[221.3, 287.8]	266.1<br>[233.5, 299.1]	295.1<br>[253.3, 338.7]	297.1<br>[275.0, 327.2]	
**70-74**	146.8<br>[97.0, 169.7]	172.3<br>[142.8, 201.2]	192.6<br>[164.8, 221.3]	210.4<br>[181.7, 242.0]	230.6<br>[201.1, 262.0]	251.2<br>[211.3, 283.2]	259.2<br>[224.8, 307.0]	291.6<br>[247.8, 337.7]	268.2<br>[248.7, 316.9]	
**75-79**	144.9<br>[118.9, 161.5]	170.4<br>[142.2, 199.1]	192.5<br>[165.1, 219.0]	206.7<br>[176.8, 239.4]	227.0<br>[196.5, 256.6]	240.8<br>[206.3, 277.0]	250.1<br>[211.3, 285.1]	260.5<br>[190.2, 281.8]		
**80-84**	148.8<br>[133.8, 170.0]	161.6<br>[132.4, 188.3]	185.1<br>[157.9, 214.0]	204.9<br>[176.2, 233.3]	220.3<br>[188.2, 252.6]	230.1<br>[197.4, 260.1]	241.9<br>[216.5, 291.7]			
**85-89**	144.1<br>[123.5, 174.9]	160.8<br>[135.8, 185.7]	182.1<br>[153.7, 207.0]	192.6<br>[164.1, 218.2]	206.7<br>[175.4, 243.4]	221.3<br>[178.9, 254.2]	242.8<br>[176.5, 272.3]			

Adrenal gland right (Male)

Age (years) \ BSA (m^2)	1.3-1.4	1.4-1.5	1.5-1.6	1.6-1.7	1.7-1.8	1.8-1.9	1.9-2.0	2.0-2.1	2.1-2.2	2.2-2.3
**20-24**		1.1 [0.8, 1.7]	1.2 [0.7, 1.8]	1.5 [1.1, 1.8]	1.6 [1.2, 1.9]	1.8 [1.5, 2.6]	2.3 [1.9, 2.7]			
**25-29**			1.2 [0.9, 1.9]	1.5 [1.0, 2.1]	1.9 [1.6, 2.5]	2.5 [1.9, 3.2]	2.6 [2.0, 2.8]	2.9 [1.9, 3.9]		
**30-34**			1.7 [1.3, 1.9]	1.8 [1.3, 2.4]	2.1 [1.5, 2.7]	2.1 [1.5, 2.5]	2.6 [2.2, 3.3]	2.7 [2.1, 3.2]	2.8 [2.6, 4.0]	
**35-39**			1.7 [1.3, 2.2]	2.1 [1.5, 2.7]	2.1 [1.6, 2.6]	2.3 [1.8, 3.1]	2.8 [2.3, 3.5]	3.2 [2.4, 3.8]	4.2 [3.2, 5.1]	
**40-44**		1.8 [1.5, 2.3]	1.5 [1.0, 1.9]	2.1 [1.5, 2.7]	2.3 [1.7, 3.1]	2.8 [2.2, 3.3]	3.3 [2.5, 3.8]	3.4 [2.9, 4.0]	3.8 [3.0, 4.3]	
**45-49**		1.7 [0.9, 2.1]	2.2 [1.4, 2.6]	2.2 [1.5, 2.7]	2.6 [2.1, 3.4]	2.9 [2.2, 3.7]	3.2 [2.5, 3.8]	3.9 [3.2, 4.7]	4.1 [3.6, 4.8]	4.5 [4.0, 5.2]
**50-54**		1.9 [1.4, 2.4]	2.1 [1.3, 2.8]	2.4 [1.8, 3.1]	2.8 [2.1, 3.4]	3.0 [2.3, 3.8]	3.3 [2.7, 4.1]	3.9 [3.0, 4.6]	4.1 [3.3, 4.9]	4.9 [4.0, 5.6]
**55-59**		1.9 [1.3, 2.6]	2.2 [1.6, 2.8]	2.6 [1.9, 3.3]	2.9 [2.3, 3.6]	3.3 [2.6, 3.9]	3.6 [2.7, 4.4]	3.9 [3.0, 4.6]	4.2 [3.3, 4.7]	4.7 [4.1, 5.3]
**60-64**	1.5 [1.3, 1.9]	2.0 [1.5, 2.7]	2.3 [1.8, 3.0]	2.7 [2.0, 3.3]	3.0 [2.4, 3.7]	3.4 [2.7, 4.0]	3.6 [2.9, 4.3]	3.7 [3.0, 4.4]	4.3 [3.3, 5.5]	4.9 [3.7, 5.5]
**65-69**	1.6 [0.8, 2.8]	1.9 [1.4, 2.5]	2.4 [1.7, 3.0]	2.8 [2.2, 3.5]	3.1 [2.5, 3.7]	3.4 [2.8, 4.1]	3.7 [3.0, 4.3]	3.8 [3.2, 4.5]	4.5 [3.9, 5.2]	4.2 [3.6, 4.7]
**70-74**	1.8 [1.1, 2.2]	1.9 [1.4, 2.6]	2.5 [1.8, 3.2]	2.8 [2.2, 3.5]	3.1 [2.6, 3.8]	3.5 [2.9, 4.1]	3.7 [3.1, 4.4]	4.0 [3.4, 4.8]	4.5 [3.6, 5.2]	
**75-79**	1.8 [1.2, 2.5]	2.2 [1.3, 2.9]	2.5 [1.9, 3.2]	2.9 [2.3, 3.6]	3.2 [2.6, 3.8]	3.4 [2.9, 4.1]	3.6 [3.0, 4.4]	3.9 [3.3, 4.5]	4.0 [3.6, 5.0]	
**80-84**	2.0 [1.2, 2.9]	2.3 [1.5, 2.9]	2.6 [2.0, 3.2]	3.0 [2.4, 3.6]	3.1 [2.5, 3.8]	3.2 [2.7, 3.8]	3.5 [2.9, 4.3]	3.9 [3.0, 4.9]		
**85-89**	2.3 [1.5, 2.6]	2.4 [1.6, 3.0]	2.6 [1.9, 3.2]	2.9 [2.3, 3.4]	3.2 [2.5, 3.9]	3.3 [2.8, 4.0]	3.6 [3.0, 4.3]			

Adrenal gland right (Female)

Age (years) \ BSA (m^2)	1.1-1.2	1.2-1.3	1.3-1.4	1.4-1.5	1.5-1.6	1.6-1.7	1.7-1.8	1.8-1.9	1.9-2.0	2.0-2.1
**20-24**		0.6 [0.4, 0.9]	1.0 [0.6, 1.4]	0.8 [0.6, 1.2]	1.1 [0.7, 1.3]	1.2 [0.8, 1.7]	1.4 [1.1, 1.8]			
**25-29**		1.1 [0.6, 1.2]	1.1 [0.8, 1.4]	0.9 [0.6, 1.2]	1.3 [0.9, 1.6]	1.4 [0.9, 1.6]	1.8 [1.2, 2.3]	1.7 [1.5, 2.2]		
**30-34**		1.1 [0.8, 1.7]	1.1 [0.7, 1.4]	1.0 [0.7, 1.4]	1.2 [0.8, 1.5]	1.4 [1.1, 2.0]	1.8 [1.3, 2.3]	1.7 [1.3, 2.4]	2.5 [2.1, 2.6]	
**35-39**		1.3 [0.7, 1.7]	1.0 [0.7, 1.3]	1.2 [0.8, 1.5]	1.3 [0.9, 1.8]	1.6 [1.3, 2.1]	2.0 [1.4, 2.4]	2.4 [1.7, 2.7]	2.5 [1.7, 2.9]	
**40-44**		0.8 [0.5, 1.1]	1.0 [0.7, 1.5]	1.2 [0.8, 1.6]	1.4 [1.0, 1.8]	1.6 [1.2, 2.1]	2.2 [1.5, 2.7]	2.5 [1.8, 3.2]	3.0 [2.6, 3.4]	3.7 [2.9, 4.4]
**45-49**		1.0 [0.4, 1.2]	1.0 [0.6, 1.5]	1.3 [0.9, 1.7]	1.5 [1.1, 2.0]	1.8 [1.4, 2.3]	2.2 [1.7, 2.9]	2.8 [2.4, 3.2]	3.3 [3.0, 3.9]	2.9 [2.6, 3.4]
**50-54**		1.0 [0.8, 1.4]	1.2 [0.7, 1.6]	1.4 [1.0, 1.8]	1.6 [1.2, 2.1]	2.1 [1.5, 2.6]	2.5 [1.9, 3.2]	2.8 [2.2, 3.6]	3.1 [2.6, 3.8]	3.5 [2.6, 3.8]
**55-59**		1.1 [0.6, 1.5]	1.1 [0.8, 1.6]	1.4 [1.0, 1.9]	1.8 [1.3, 2.3]	2.2 [1.7, 2.7]	2.7 [2.2, 3.4]	2.9 [2.4, 3.5]	3.2 [2.9, 3.7]	
**60-64**	1.1 [0.7, 1.7]	1.1 [0.6, 1.5]	1.4 [0.9, 1.7]	1.6 [1.1, 2.1]	1.9 [1.4, 2.5]	2.3 [1.8, 3.0]	2.8 [2.3, 3.3]	2.9 [2.3, 3.5]	3.6 [2.8, 3.9]	3.6 [3.4, 4.3]
**65-69**	1.0 [0.5, 1.6]	1.2 [0.8, 1.6]	1.5 [0.9, 1.9]	1.8 [1.2, 2.4]	2.1 [1.6, 2.8]	2.5 [2.0, 3.1]	2.9 [2.4, 3.5]	3.4 [2.6, 3.9]	3.4 [3.0, 4.1]	
**70-74**	1.2 [0.7, 1.6]	1.2 [0.7, 1.8]	1.6 [1.1, 2.2]	2.0 [1.4, 2.5]	2.3 [1.7, 2.9]	2.8 [2.2, 3.4]	2.9 [2.3, 3.5]	3.3 [2.5, 3.6]	3.3 [2.4, 4.1]	
**75-79**	1.2 [0.7, 1.9]	1.4 [0.9, 2.0]	1.8 [1.1, 2.3]	2.1 [1.5, 2.6]	2.4 [1.9, 3.1]	2.9 [2.2, 3.5]	3.0 [2.5, 3.5]	3.2 [2.5, 3.9]		
**80-84**	1.1 [0.7, 1.6]	1.5 [0.9, 2.1]	1.8 [1.3, 2.4]	2.2 [1.7, 2.8]	2.5 [1.9, 3.1]	2.9 [2.4, 3.4]	3.2 [2.3, 3.8]			
**85-89**	1.3 [0.4, 1.8]	1.5 [0.8, 2.1]	2.0 [1.5, 2.5]	2.2 [1.8, 2.8]	2.5 [2.1, 3.1]	2.8 [2.1, 3.3]	3.5 [2.9, 4.5]			

Adrenal gland left (Male)

Age (years) \ BSA (m^2)	1.3-1.4	1.4-1.5	1.5-1.6	1.6-1.7	1.7-1.8	1.8-1.9	1.9-2.0	2.0-2.1	2.1-2.2	2.2-2.3
**20-24**		1.7<br>[0.9, 2.4]	1.5<br>[1.0, 2.5]	2.0<br>[1.2, 2.6]	2.1<br>[1.5, 2.4]	2.5<br>[1.7, 3.1]	3.3<br>[2.9, 3.4]			
**25-29**			1.8<br>[1.2, 2.5]	2.2<br>[1.7, 3.0]	2.5<br>[2.1, 3.3]	3.2<br>[2.6, 4.3]	2.9<br>[2.1, 3.4]	3.7<br>[2.8, 4.3]		
**30-34**			2.2<br>[1.7, 2.5]	2.4<br>[1.9, 3.0]	3.0<br>[2.3, 3.4]	3.2<br>[2.3, 3.7]	3.6<br>[2.7, 4.1]	3.7<br>[3.2, 4.5]	4.6<br>[4.3, 5.0]	
**35-39**			2.1<br>[1.6, 2.8]	2.6<br>[1.8, 3.5]	3.1<br>[2.4, 3.9]	3.2<br>[2.5, 4.1]	3.6<br>[2.8, 4.5]	4.1<br>[3.3, 4.8]	4.5<br>[4.0, 5.2]	
**40-44**		2.6<br>[1.2, 3.1]	2.3<br>[1.6, 2.9]	2.7<br>[1.9, 3.5]	3.2<br>[2.3, 3.8]	3.7<br>[2.9, 4.4]	4.1<br>[3.6, 4.9]	4.8<br>[3.8, 5.6]	5.0<br>[4.0, 5.6]	
**45-49**		1.9<br>[1.2, 2.8]	2.8<br>[2.1, 3.6]	2.8<br>[2.1, 3.5]	3.4<br>[2.8, 4.2]	3.6<br>[3.0, 4.4]	4.1<br>[3.4, 4.9]	4.4<br>[3.8, 5.6]	5.0<br>[4.0, 6.1]	6.1<br>[5.0, 7.0]
**50-54**		2.6<br>[1.8, 3.1]	2.8<br>[2.1, 3.4]	3.1<br>[2.3, 3.8]	3.5<br>[2.8, 4.2]	4.0<br>[3.1, 4.9]	4.5<br>[3.5, 5.2]	4.9<br>[4.0, 5.8]	5.4<br>[4.7, 6.0]	5.9<br>[5.0, 6.7]
**55-59**		2.5<br>[1.5, 3.0]	2.9<br>[2.2, 3.8]	3.3<br>[2.7, 4.2]	3.9<br>[3.1, 4.6]	4.2<br>[3.4, 5.0]	4.6<br>[3.6, 5.5]	4.7<br>[4.0, 5.8]	5.3<br>[4.3, 6.2]	5.9<br>[5.0, 7.2]
**60-64**	2.0<br>[1.8, 2.7]	2.8<br>[1.9, 3.6]	3.1<br>[2.0, 3.8]	3.6<br>[2.7, 4.3]	3.8<br>[3.1, 4.6]	4.3<br>[3.5, 5.2]	4.6<br>[3.8, 5.6]	4.8<br>[3.9, 5.9]	5.4<br>[4.1, 6.5]	6.0<br>[5.3, 7.5]
**65-69**	2.2<br>[1.4, 3.1]	2.6<br>[1.8, 3.4]	3.1<br>[2.1, 3.8]	3.6<br>[2.8, 4.2]	3.9<br>[3.2, 4.7]	4.3<br>[3.5, 5.2]	4.6<br>[3.7, 5.5]	4.9<br>[3.9, 6.1]	5.3<br>[4.8, 6.9]	4.9<br>[4.5, 6.3]
**70-74**	2.3<br>[1.5, 2.6]	2.6<br>[1.7, 3.4]	3.2<br>[2.3, 3.9]	3.5<br>[2.8, 4.3]	3.9<br>[3.2, 4.8]	4.3<br>[3.6, 5.3]	4.7<br>[3.8, 5.5]	4.9<br>[4.3, 5.6]	5.1<br>[4.0, 5.8]	
**75-79**	2.2<br>[1.7, 3.0]	2.8<br>[2.0, 3.6]	3.2<br>[2.4, 4.0]	3.7<br>[2.9, 4.5]	4.0<br>[3.2, 4.8]	4.3<br>[3.5, 5.1]	4.7<br>[3.8, 5.6]	5.1<br>[4.2, 6.2]	5.2<br>[4.5, 7.1]	
**80-84**	2.3<br>[1.4, 3.1]	2.8<br>[2.0, 3.6]	3.2<br>[2.5, 4.0]	3.6<br>[2.9, 4.4]	4.0<br>[3.3, 4.8]	4.3<br>[3.5, 5.0]	4.3<br>[3.6, 5.1]	4.6<br>[4.0, 5.2]		
**85-89**	2.8<br>[2.2, 3.3]	2.8<br>[1.9, 3.6]	3.2<br>[2.4, 4.0]	3.6<br>[2.8, 4.2]	4.0<br>[3.1, 4.8]	4.3<br>[3.4, 4.8]	4.9<br>[4.0, 5.3]			

Adrenal gland left (Female)

Age (years) \ BSA (m^2)	1.1-1.2	1.2-1.3	1.3-1.4	1.4-1.5	1.5-1.6	1.6-1.7	1.7-1.8	1.8-1.9	1.9-2.0	2.0-2.1
**20-24**		1.4<br>[0.8, 2.2]	1.3<br>[0.9, 1.6]	1.3<br>[0.9, 1.9]	1.5<br>[1.0, 1.9]	1.6<br>[0.9, 2.2]	2.0<br>[1.6, 2.6]			
**25-29**		0.9<br>[0.6, 1.8]	1.4<br>[1.0, 1.7]	1.4<br>[0.9, 2.0]	1.7<br>[1.2, 2.4]	1.8<br>[1.3, 2.4]	2.6<br>[2.1, 3.3]	2.4<br>[1.9, 2.9]		
**30-34**			1.8<br>[1.4, 2.3]	1.6<br>[1.0, 2.0]	1.7<br>[1.1, 2.2]	2.0<br>[1.5, 2.5]	2.7<br>[1.9, 3.3]	2.9<br>[2.4, 3.4]	3.2<br>[2.3, 4.2]	
**35-39**		1.9<br>[1.2, 2.2]	1.4<br>[0.4, 1.8]	1.6<br>[1.1, 2.2]	1.8<br>[1.3, 2.3]	2.1<br>[1.7, 2.6]	2.7<br>[2.0, 3.1]	2.9<br>[2.5, 3.5]	3.4<br>[2.9, 4.1]	
**40-44**		0.6<br>[0.2, 1.6]	1.6<br>[1.0, 2.0]	1.6<br>[1.0, 2.2]	2.0<br>[1.4, 2.6]	2.3<br>[1.6, 2.9]	2.8<br>[2.2, 3.5]	3.1<br>[2.5, 3.7]	3.8<br>[3.3, 4.5]	4.9<br>[4.1, 5.2]
**45-49**		1.4<br>[0.7, 1.9]	1.5<br>[1.1, 2.0]	1.8<br>[1.2, 2.4]	2.2<br>[1.6, 2.7]	2.5<br>[1.8, 3.1]	3.0<br>[2.3, 3.6]	3.6<br>[2.9, 4.4]	4.1<br>[3.4, 5.0]	3.6<br>[3.2, 4.6]
**50-54**		1.5<br>[0.8, 2.5]	1.8<br>[1.1, 2.3]	1.9<br>[1.2, 2.5]	2.3<br>[1.6, 2.8]	2.7<br>[2.0, 3.4]	3.2<br>[2.3, 3.9]	3.6<br>[3.1, 4.2]	3.8<br>[3.0, 4.6]	4.6<br>[3.9, 5.5]
**55-59**		1.3<br>[0.5, 2.1]	1.6<br>[1.0, 2.3]	2.0<br>[1.4, 2.6]	2.4<br>[1.8, 3.1]	2.8<br>[2.2, 3.5]	3.5<br>[2.7, 4.2]	3.6<br>[2.8, 4.5]	4.4<br>[3.6, 4.9]	
**60-64**	1.4<br>[1.0, 1.7]	1.5<br>[0.8, 2.1]	1.7<br>[1.1, 2.4]	2.2<br>[1.6, 2.8]	2.6<br>[1.9, 3.3]	3.0<br>[2.3, 3.8]	3.4<br>[2.8, 4.1]	3.8<br>[3.0, 4.6]	4.3<br>[3.8, 5.1]	4.4<br>[3.6, 6.1]
**65-69**	1.4<br>[0.6, 1.7]	1.6<br>[0.8, 2.3]	1.9<br>[1.1, 2.5]	2.3<br>[1.6, 3.0]	2.8<br>[2.2, 3.5]	3.1<br>[2.5, 3.8]	3.6<br>[3.1, 4.3]	3.9<br>[3.1, 5.0]	4.6<br>[3.9, 5.4]	
**70-74**	1.3<br>[0.4, 2.1]	1.8<br>[1.0, 2.4]	2.1<br>[1.4, 2.8]	2.4<br>[1.7, 3.1]	2.9<br>[2.3, 3.6]	3.3<br>[2.7, 4.0]	3.6<br>[2.8, 4.3]	4.2<br>[3.0, 5.0]	3.4<br>[2.4, 4.3]	
**75-79**	1.5<br>[0.6, 2.3]	1.8<br>[1.0, 2.3]	2.1<br>[1.5, 2.7]	2.5<br>[1.8, 3.2]	3.0<br>[2.3, 3.7]	3.5<br>[2.7, 4.1]	3.6<br>[2.9, 4.3]	4.3<br>[3.8, 5.0]		
**80-84**	1.3<br>[0.9, 2.2]	1.8<br>[0.9, 2.6]	2.3<br>[1.6, 2.9]	2.6<br>[2.0, 3.3]	3.0<br>[2.4, 3.7]	3.4<br>[2.8, 4.2]	4.0<br>[3.1, 4.6]			
**85-89**	1.4<br>[0.8, 2.1]	1.9<br>[1.1, 2.7]	2.4<br>[1.5, 3.1]	2.8<br>[2.0, 3.4]	3.2<br>[2.5, 3.8]	3.4<br>[2.6, 4.0]	3.9<br>[3.4, 5.3]			

Adrenal gland total (Male)

Age (years) \ BSA (m^2)	1.3-1.4	1.4-1.5	1.5-1.6	1.6-1.7	1.7-1.8	1.8-1.9	1.9-2.0	2.0-2.1	2.1-2.2	2.2-2.3
**20-24**		2.8<br>[1.7, 4.0]	2.9<br>[1.7, 4.0]	3.1<br>[2.5, 4.2]	3.5<br>[2.9, 4.2]	4.3<br>[2.9, 5.4]	5.6<br>[4.5, 6.0]			
**25-29**			3.2<br>[2.5, 4.2]	3.6<br>[2.5, 4.7]	4.3<br>[3.7, 5.9]	5.4<br>[4.5, 7.7]	5.6<br>[4.2, 6.2]	6.6<br>[5.2, 8.2]		
**30-34**			3.8<br>[3.2, 4.5]	4.3<br>[3.4, 5.3]	4.8<br>[3.5, 5.9]	4.9<br>[4.1, 5.9]	6.2<br>[4.7, 7.4]	6.4<br>[5.6, 7.7]	7.4<br>[6.2, 9.0]	
**35-39**			3.7<br>[2.5, 4.8]	4.3<br>[3.5, 5.6]	5.2<br>[4.0, 6.2]	5.5<br>[4.1, 6.8]	6.3<br>[5.3, 7.8]	7.2<br>[6.2, 8.6]	9.0<br>[7.2, 10.3]	
**40-44**		3.6<br>[2.4, 5.7]	3.5<br>[2.7, 4.7]	4.9<br>[3.6, 6.1]	5.3<br>[4.2, 6.7]	6.3<br>[5.3, 7.7]	7.5<br>[6.1, 8.6]	8.6<br>[6.5, 9.4]	8.2<br>[7.2, 9.5]	
**45-49**		3.2<br>[2.5, 4.3]	4.6<br>[3.5, 5.9]	4.8<br>[3.5, 6.0]	6.0<br>[4.8, 7.3]	6.3<br>[5.3, 8.1]	7.2<br>[5.8, 8.4]	8.2<br>[7.0, 10.0]	8.8<br>[7.3, 11.1]	10.2<br>[9.5, 12.5]
**50-54**		4.4<br>[3.3, 5.3]	4.7<br>[3.2, 6.1]	5.6<br>[4.0, 6.9]	6.3<br>[4.9, 7.5]	6.8<br>[5.4, 8.2]	7.8<br>[6.4, 9.0]	8.7<br>[7.2, 10.0]	9.3<br>[8.0, 10.7]	10.4<br>[9.0, 12.5]
**55-59**		4.0<br>[3.0, 5.6]	5.1<br>[3.6, 6.5]	6.0<br>[4.6, 7.3]	6.7<br>[5.4, 8.1]	7.4<br>[6.0, 8.7]	8.1<br>[6.5, 9.7]	8.4<br>[7.4, 9.8]	9.4<br>[8.5, 11.0]	10.8<br>[8.4, 12.1]
**60-64**	3.8<br>[3.3, 4.2]	4.6<br>[3.0, 6.1]	5.3<br>[3.8, 6.6]	6.2<br>[4.8, 7.4]	6.8<br>[5.6, 8.1]	7.6<br>[6.5, 9.2]	8.1<br>[6.9, 9.8]	8.4<br>[7.2, 9.9]	9.7<br>[7.2, 11.2]	11.1<br>[8.5, 12.4]
**65-69**	3.6<br>[2.0, 5.3]	4.4<br>[3.4, 5.7]	5.3<br>[3.8, 6.6]	6.2<br>[5.1, 7.5]	7.0<br>[5.7, 8.1]	7.7<br>[6.3, 9.1]	8.2<br>[6.8, 9.6]	8.6<br>[7.2, 10.4]	9.7<br>[8.5, 12.6]	9.4<br>[7.9, 10.6]
**70-74**	4.2<br>[2.2, 4.7]	4.3<br>[3.2, 5.8]	5.6<br>[4.1, 6.8]	6.3<br>[5.1, 7.6]	7.0<br>[5.8, 8.5]	7.8<br>[6.6, 9.1]	8.4<br>[6.9, 9.8]	9.1<br>[7.8, 10.3]	9.0<br>[7.9, 11.1]	
**75-79**	4.0<br>[2.7, 5.1]	4.8<br>[3.2, 6.3]	5.7<br>[4.4, 6.9]	6.6<br>[5.2, 7.9]	7.1<br>[5.9, 8.4]	7.6<br>[6.5, 9.1]	8.2<br>[6.8, 9.7]	9.1<br>[7.5, 10.6]	9.2<br>[7.6, 11.7]	
**80-84**	4.1<br>[2.5, 5.4]	4.9<br>[3.3, 6.2]	5.7<br>[4.5, 7.0]	6.5<br>[5.3, 7.7]	7.1<br>[5.8, 8.4]	7.5<br>[6.4, 8.5]	7.9<br>[6.7, 8.8]	7.9<br>[7.0, 10.2]		
**85-89**	4.8<br>[3.8, 5.7]	4.8<br>[3.8, 6.2]	5.7<br>[4.3, 7.0]	6.4<br>[5.2, 7.4]	6.9<br>[5.7, 8.4]	7.4<br>[6.2, 8.8]	8.5<br>[6.6, 9.7]			

Adrenal gland total (Female)

Age (years) \ BSA (m^2)	1.1-1.2	1.2-1.3	1.3-1.4	1.4-1.5	1.5-1.6	1.6-1.7	1.7-1.8	1.8-1.9	1.9-2.0	2.0-2.1
**20-24**		1.8<br>[0.7, 3.0]	2.2<br>[1.6, 2.6]	2.1<br>[1.3, 2.6]	2.5<br>[1.6, 3.0]	2.7<br>[2.1, 3.6]	3.4<br>[2.6, 4.2]			
**25-29**		1.8<br>[1.1, 3.2]	2.3<br>[1.7, 3.0]	2.2<br>[1.2, 2.9]	3.0<br>[2.3, 3.8]	3.1<br>[2.3, 3.6]	4.8<br>[3.1, 5.5]	4.1<br>[3.6, 5.1]		
**30-34**		2.1<br>[1.5, 3.1]	2.8<br>[1.9, 3.5]	2.5<br>[1.7, 3.1]	2.7<br>[2.0, 3.5]	3.4<br>[2.7, 4.2]	4.4<br>[3.1, 5.3]	4.6<br>[3.9, 5.4]	5.6<br>[4.7, 6.5]	
**35-39**		3.1<br>[1.9, 3.8]	2.1<br>[1.3, 3.0]	2.8<br>[2.1, 3.6]	2.9<br>[2.2, 4.0]	3.6<br>[3.0, 4.5]	4.6<br>[3.6, 5.5]	5.3<br>[4.5, 6.1]	6.3<br>[4.9, 6.7]	
**40-44**		1.1<br>[0.6, 2.6]	2.5<br>[1.7, 3.2]	2.7<br>[1.8, 3.5]	3.4<br>[2.5, 4.2]	3.8<br>[2.8, 4.9]	4.8<br>[3.8, 6.1]	5.4<br>[4.3, 6.7]	6.8<br>[5.6, 7.6]	8.0<br>[7.3, 9.0]
**45-49**		2.1<br>[1.5, 2.8]	2.5<br>[1.6, 3.2]	3.0<br>[2.2, 3.9]	3.7<br>[2.8, 4.6]	4.1<br>[3.1, 5.2]	5.1<br>[4.2, 6.3]	6.3<br>[5.5, 7.4]	7.2<br>[6.1, 8.6]	6.7<br>[5.7, 7.9]
**50-54**		2.2<br>[1.2, 3.4]	2.8<br>[1.9, 3.7]	3.2<br>[2.3, 4.0]	3.8<br>[2.9, 4.8]	4.7<br>[3.6, 5.9]	5.7<br>[4.4, 7.0]	6.5<br>[5.4, 7.8]	7.0<br>[5.9, 8.1]	8.1<br>[6.5, 9.7]
**55-59**		1.8<br>[0.9, 3.0]	2.7<br>[1.7, 3.6]	3.3<br>[2.4, 4.4]	4.1<br>[3.2, 5.2]	4.9<br>[3.9, 6.1]	6.1<br>[4.9, 7.5]	6.6<br>[5.2, 7.8]	7.6<br>[6.5, 8.7]	
**60-64**	2.5<br>[0.8, 3.0]	2.5<br>[1.4, 3.4]	2.9<br>[1.9, 3.9]	3.7<br>[2.8, 4.7]	4.5<br>[3.4, 5.7]	5.4<br>[4.3, 6.5]	6.3<br>[5.1, 7.4]	6.7<br>[5.5, 7.8]	7.7<br>[7.0, 9.0]	8.1<br>[7.0, 10.2]
**65-69**	2.1<br>[1.3, 3.1]	2.6<br>[1.5, 3.7]	3.1<br>[2.0, 4.2]	4.0<br>[3.0, 5.2]	4.9<br>[3.8, 6.0]	5.7<br>[4.6, 6.7]	6.5<br>[5.6, 7.6]	7.4<br>[6.1, 8.8]	8.0<br>[7.0, 9.8]	
**70-74**	2.3<br>[1.0, 3.6]	2.8<br>[1.6, 3.9]	3.5<br>[2.3, 4.7]	4.3<br>[3.1, 5.5]	5.2<br>[4.1, 6.3]	6.1<br>[4.9, 7.3]	6.4<br>[5.2, 7.7]	7.2<br>[5.4, 8.7]	6.3<br>[4.9, 8.5]	
**75-79**	2.2<br>[1.5, 3.9]	2.9<br>[1.8, 4.0]	3.7<br>[2.5, 4.8]	4.6<br>[3.4, 5.6]	5.4<br>[4.3, 6.6]	6.4<br>[4.9, 7.4]	6.5<br>[5.7, 7.7]	7.3<br>[6.0, 8.4]		
**80-84**	2.0<br>[1.2, 3.4]	2.8<br>[1.7, 4.3]	3.9<br>[2.6, 5.1]	4.7<br>[3.7, 5.9]	5.5<br>[4.4, 6.6]	6.3<br>[5.5, 7.4]	6.7<br>[5.9, 8.2]			
**85-89**	2.1<br>[0.9, 3.6]	3.2<br>[1.8, 4.5]	4.1<br>[3.0, 5.3]	5.0<br>[3.8, 5.9]	5.7<br>[4.6, 6.8]	5.9<br>[4.9, 7.1]	6.9<br>[6.3, 9.1]			

Prostate (Male)

**Age (years) \ BSA (m^2)**	**1.3-1.4**	**1.4-1.5**	**1.5-1.6**	**1.6-1.7**	**1.7-1.8**	**1.8-1.9**	**1.9-2.0**	**2.0-2.1**	**2.1-2.2**	**2.2-2.3**
**20-24**		20.2<br>[15.8, 22.2]	21.0<br>[16.8, 24.3]	22.2<br>[18.4, 25.8]	21.7<br>[18.8, 23.8]	22.8<br>[20.7, 27.1]	25.0<br>[20.7, 26.8]			
**25-29**			21.5<br>[19.5, 23.2]	20.2<br>[17.0, 24.2]	22.4<br>[20.3, 24.2]	23.6<br>[20.8, 25.4]	24.1<br>[20.1, 26.3]	21.8<br>[19.7, 26.7]		
**30-34**			20.1<br>[17.3, 24.2]	22.1<br>[19.5, 24.2]	24.7<br>[21.1, 28.2]	23.6<br>[20.2, 26.1]	25.6<br>[20.7, 28.7]	27.4<br>[24.5, 28.6]	28.8<br>[23.9, 33.3]	
**35-39**			21.7<br>[17.0, 24.3]	21.8<br>[20.2, 25.7]	23.3<br>[20.8, 26.5]	25.1<br>[21.6, 28.6]	24.9<br>[22.0, 27.8]	25.4<br>[21.4, 27.2]	27.0<br>[24.8, 33.1]	
**40-44**		20.6<br>[15.8, 23.8]	21.1<br>[17.3, 25.0]	22.4<br>[20.2, 26.5]	24.3<br>[21.5, 27.6]	24.6<br>[21.2, 28.6]	26.7<br>[23.4, 29.9]	25.0<br>[22.6, 28.9]	29.1<br>[25.4, 30.8]	
**45-49**		22.6<br>[16.4, 23.8]	20.5<br>[18.4, 25.7]	23.6<br>[20.6, 27.5]	24.7<br>[21.5, 28.1]	25.0<br>[22.3, 29.5]	27.5<br>[23.3, 31.3]	25.9<br>[23.0, 29.2]	30.4<br>[26.6, 33.7]	28.8<br>[26.6, 32.4]
**50-54**		19.8<br>[17.0, 22.0]	21.5<br>[18.6, 25.2]	23.6<br>[20.4, 27.2]	25.3<br>[21.4, 28.6]	25.9<br>[22.7, 29.1]	27.4<br>[23.4, 30.4]	28.3<br>[24.8, 31.6]	29.0<br>[23.5, 33.5]	32.2<br>[28.5, 34.8]
**55-59**		19.2<br>[15.2, 22.2]	22.0<br>[18.6, 26.2]	23.7<br>[19.8, 27.5]	25.9<br>[22.5, 29.5]	26.8<br>[22.9, 30.3]	28.0<br>[23.4, 31.7]	28.3<br>[24.7, 32.9]	29.6<br>[25.6, 36.6]	31.4<br>[28.2, 37.9]
**60-64**	16.7<br>[14.3, 18.4]	19.4<br>[16.2, 23.4]	22.8<br>[18.5, 25.5]	24.3<br>[20.8, 28.7]	25.9<br>[21.8, 30.1]	27.8<br>[23.1, 32.8]	28.9<br>[23.7, 33.3]	30.3<br>[25.7, 34.3]	30.2<br>[26.4, 35.5]	38.1<br>[29.0, 40.8]
**65-69**	16.4<br>[13.4, 21.9]	20.2<br>[16.1, 23.6]	22.6<br>[18.6, 26.6]	24.7<br>[20.1, 29.1]	26.5<br>[22.3, 31.1]	28.0<br>[22.6, 32.9]	29.4<br>[23.9, 35.3]	30.7<br>[25.3, 35.4]	29.6<br>[26.1, 34.9]	30.0<br>[28.5, 42.8]
**70-74**	19.3<br>[13.6, 23.6]	21.3<br>[17.8, 25.3]	23.1<br>[18.6, 27.9]	25.2<br>[20.8, 31.0]	26.2<br>[21.2, 31.9]	27.6<br>[23.0, 33.3]	29.4<br>[24.1, 35.2]	30.8<br>[25.7, 37.8]	30.2<br>[26.8, 42.6]	
**75-79**	18.5<br>[14.9, 23.6]	21.7<br>[17.6, 26.6]	23.1<br>[18.6, 27.6]	25.1<br>[19.8, 31.1]	26.9<br>[20.8, 32.5]	29.1<br>[23.0, 35.6]	29.1<br>[22.9, 37.6]	30.9<br>[24.9, 40.5]	34.0<br>[24.9, 41.8]	
**80-84**	19.0<br>[12.9, 25.1]	21.4<br>[17.1, 26.0]	23.2<br>[18.0, 28.9]	24.6<br>[19.3, 30.9]	25.7<br>[20.7, 33.4]	28.7<br>[22.8, 35.5]	28.5<br>[20.1, 36.4]	29.6<br>[23.8, 33.8]		
**85-89**	17.5<br>[12.2, 22.8]	21.2<br>[16.1, 26.6]	23.4<br>[16.9, 29.3]	25.3<br>[19.1, 32.1]	26.5<br>[19.9, 33.5]	27.8<br>[22.0, 37.8]	26.9<br>[23.5, 33.7]			

Iliopsoas muscle right (Male)

Age (years) \ BSA (m^2)	1.3-1.4	1.4-1.5	1.5-1.6	1.6-1.7	1.7-1.8	1.8-1.9	1.9-2.0	2.0-2.1	2.1-2.2	2.2-2.3
**20-24**		223.0<br>[212.1, 262.9]	278.7<br>[238.5, 316.0]	301.6<br>[270.6, 341.3]	313.8<br>[286.6, 350.2]	348.8<br>[295.2, 389.0]	361.9<br>[351.6, 396.3]			
**25-29**			272.4<br>[220.5, 284.1]	296.9<br>[273.7, 327.6]	308.0<br>[267.1, 339.5]	340.2<br>[309.3, 376.7]	371.5<br>[347.0, 421.4]	372.2<br>[302.3, 403.3]		
**30-34**			249.1<br>[225.4, 287.7]	277.4<br>[242.6, 307.4]	302.9<br>[269.9, 332.1]	323.5<br>[299.6, 362.7]	349.4<br>[315.5, 391.9]	375.9<br>[319.1, 382.9]	402.8<br>[370.5, 427.2]	
**35-39**			256.2<br>[232.9, 289.4]	279.2<br>[246.5, 307.6]	297.6<br>[260.6, 322.6]	329.4<br>[299.3, 357.0]	354.3<br>[317.0, 374.9]	344.5<br>[308.4, 376.2]	380.1<br>[350.7, 461.4]	
**40-44**		192.3<br>[139.2, 212.2]	238.0<br>[184.3, 278.4]	268.8<br>[237.4, 296.6]	292.0<br>[269.0, 326.2]	321.7<br>[288.2, 349.3]	339.2<br>[311.1, 365.7]	369.0<br>[315.5, 401.6]	371.1<br>[342.0, 399.2]	
**45-49**		204.8<br>[185.4, 236.0]	220.1<br>[186.8, 256.5]	261.1<br>[228.1, 285.8]	287.1<br>[257.0, 317.1]	306.8<br>[271.0, 338.1]	331.2<br>[295.7, 360.8]	340.6<br>[300.9, 371.6]	368.5<br>[331.8, 402.3]	368.2<br>[322.9, 406.9]
**50-54**		207.9<br>[180.3, 231.8]	218.2<br>[187.5, 244.9]	247.8<br>[219.2, 269.7]	272.5<br>[244.8, 299.6]	297.8<br>[265.7, 323.2]	322.2<br>[283.8, 353.9]	324.2<br>[291.1, 361.1]	361.9<br>[314.8, 394.1]	364.5<br>[317.9, 425.3]
**55-59**		191.6<br>[169.8, 228.8]	220.6<br>[193.0, 238.6]	241.2<br>[208.4, 265.7]	260.8<br>[230.3, 287.7]	288.0<br>[258.0, 316.2]	301.5<br>[265.1, 330.7]	325.1<br>[287.8, 353.2]	346.2<br>[317.7, 373.7]	346.2<br>[332.9, 383.1]
**60-64**	169.5<br>[148.2, 177.5]	191.4<br>[164.8, 208.8]	205.8<br>[178.5, 231.2]	237.6<br>[208.7, 260.7]	258.4<br>[229.6, 282.1]	275.2<br>[244.9, 302.6]	294.8<br>[263.0, 320.1]	304.0<br>[273.2, 337.5]	334.7<br>[284.2, 365.9]	334.4<br>[319.9, 366.9]
**65-69**	156.9<br>[140.6, 190.1]	185.0<br>[168.0, 212.2]	201.6<br>[179.5, 229.2]	231.5<br>[204.5, 258.6]	246.9<br>[217.6, 274.2]	260.4<br>[228.9, 288.8]	282.4<br>[253.2, 315.2]	300.0<br>[268.4, 328.1]	311.3<br>[270.5, 346.3]	350.7<br>[334.4, 394.1]
**70-74**	159.4<br>[141.6, 181.8]	176.8<br>[153.1, 197.1]	201.1<br>[176.0, 222.8]	218.8<br>[193.3, 243.2]	238.1<br>[212.4, 262.8]	252.7<br>[228.4, 282.2]	272.1<br>[241.7, 303.1]	303.4<br>[264.8, 332.4]	311.3<br>[284.1, 341.2]	
**75-79**	153.1<br>[134.5, 171.6]	179.6<br>[158.3, 200.3]	197.3<br>[170.1, 219.5]	212.1<br>[185.5, 235.0]	230.0<br>[205.1, 255.6]	247.5<br>[219.4, 275.6]	265.3<br>[238.4, 295.0]	273.2<br>[250.9, 307.5]	293.1<br>[262.4, 319.6]	
**80-84**	156.5<br>[134.5, 172.3]	174.1<br>[155.0, 192.5]	190.6<br>[169.4, 212.9]	209.4<br>[187.3, 231.5]	225.5<br>[201.5, 247.9]	243.1<br>[217.3, 270.2]	263.4<br>[233.1, 282.9]	268.2<br>[216.2, 307.5]		
**85-89**	167.0<br>[141.2, 179.7]	172.0<br>[148.1, 189.4]	190.1<br>[169.1, 209.0]	203.0<br>[180.8, 228.1]	213.4<br>[190.6, 237.6]	231.8<br>[202.1, 258.2]	253.8<br>[234.5, 277.6]			

Iliopsoas muscle right (Female)

Age (years) \ BSA (m^2)	1.1-1.2	1.2-1.3	1.3-1.4	1.4-1.5	1.5-1.6	1.6-1.7	1.7-1.8	1.8-1.9	1.9-2.0	2.0-2.1
**20-24**		119.0<br>[114.4, 128.0]	159.3<br>[138.1, 172.5]	182.1<br>[167.3, 198.9]	196.8<br>[177.4, 212.1]	208.0<br>[186.3, 219.2]	232.5<br>[197.7, 245.3]			
**25-29**		126.4<br>[112.0, 143.2]	153.8<br>[137.8, 163.2]	180.0<br>[161.0, 195.3]	187.9<br>[169.9, 207.1]	192.7<br>[171.6, 210.3]	205.4<br>[190.3, 227.1]	226.0<br>[206.7, 243.7]		
**30-34**		110.8<br>[93.7, 131.0]	159.0<br>[138.6, 166.3]	173.1<br>[155.4, 189.1]	186.7<br>[168.7, 201.1]	205.6<br>[188.9, 219.5]	200.0<br>[186.5, 221.0]	214.4<br>[204.9, 234.4]	275.6<br>[243.6, 308.8]	
**35-39**		126.4<br>[101.7, 138.2]	154.6<br>[141.4, 171.6]	176.7<br>[159.7, 192.1]	185.3<br>[165.0, 205.6]	197.4<br>[182.1, 216.9]	222.2<br>[195.6, 241.1]	225.3<br>[201.2, 235.8]	213.6<br>[183.0, 238.1]	
**40-44**		127.8<br>[104.5, 142.6]	153.2<br>[138.3, 174.4]	171.6<br>[152.9, 188.5]	182.4<br>[167.6, 198.2]	197.0<br>[178.1, 214.9]	209.8<br>[190.5, 224.2]	226.1<br>[201.0, 247.3]	229.6<br>[207.5, 254.8]	242.3<br>[224.3, 266.4]
**45-49**		138.6<br>[123.3, 147.6]	151.0<br>[135.4, 166.9]	163.2<br>[147.4, 181.5]	177.0<br>[160.2, 194.4]	189.6<br>[170.8, 202.7]	201.2<br>[185.2, 218.9]	211.3<br>[193.1, 237.6]	216.5<br>[202.9, 244.8]	240.6<br>[218.9, 247.8]
**50-54**		125.9<br>[106.7, 139.7]	144.6<br>[124.3, 161.9]	160.2<br>[144.2, 177.5]	173.2<br>[155.3, 188.2]	183.5<br>[164.8, 200.3]	191.6<br>[174.6, 213.9]	199.6<br>[177.7, 230.0]	221.5<br>[204.8, 241.9]	223.6<br>[203.7, 235.4]
**55-59**		126.4<br>[105.2, 143.7]	145.7<br>[127.5, 159.7]	156.7<br>[138.8, 172.5]	167.7<br>[149.5, 181.6]	176.7<br>[160.9, 193.7]	190.5<br>[168.9, 210.6]	205.1<br>[178.8, 230.3]	213.9<br>[194.6, 241.0]	
**60-64**	117.5<br>[113.6, 140.0]	129.2<br>[113.7, 148.0]	140.7<br>[123.4, 152.6]	153.8<br>[138.2, 169.0]	163.2<br>[144.1, 181.0]	173.7<br>[155.2, 194.0]	180.2<br>[168.3, 199.7]	197.7<br>[181.5, 216.4]	207.6<br>[190.2, 224.2]	224.0<br>[192.7, 234.9]
**65-69**	114.6<br>[98.4, 127.9]	122.9<br>[109.1, 136.9]	137.9<br>[124.8, 153.8]	149.0<br>[131.0, 164.2]	160.2<br>[141.9, 176.3]	169.8<br>[151.1, 188.4]	182.2<br>[164.8, 201.1]	190.3<br>[176.3, 213.0]	197.3<br>[185.8, 214.9]	
**70-74**	109.8<br>[101.1, 125.1]	122.6<br>[107.3, 136.2]	134.9<br>[117.6, 150.6]	146.7<br>[128.3, 161.4]	156.0<br>[138.9, 173.4]	165.5<br>[147.7, 184.8]	182.4<br>[160.1, 198.8]	190.5<br>[162.3, 207.7]	183.5<br>[166.3, 218.5]	
**75-79**	116.2<br>[101.0, 127.8]	125.1<br>[109.3, 139.5]	132.1<br>[115.2, 147.0]	142.1<br>[125.9, 158.7]	154.5<br>[134.4, 170.5]	164.9<br>[149.0, 182.3]	179.5<br>[161.4, 192.1]	172.2<br>[150.7, 188.2]		
**80-84**	114.6<br>[92.5, 133.6]	124.7<br>[111.0, 141.2]	130.3<br>[115.2, 146.5]	143.0<br>[126.3, 158.6]	155.1<br>[136.1, 169.5]	159.9<br>[142.3, 180.0]	164.1<br>[144.5, 195.5]			
**85-89**	105.5<br>[86.5, 123.8]	123.3<br>[107.6, 138.3]	130.9<br>[111.4, 146.2]	141.2<br>[123.9, 157.6]	149.7<br>[129.9, 165.2]	162.0<br>[141.8, 175.1]	167.8<br>[162.1, 181.9]			

Iliopsoas muscle left (Male)

Age (years) \ BSA (m^2)	1.3-1.4	1.4-1.5	1.5-1.6	1.6-1.7	1.7-1.8	1.8-1.9	1.9-2.0	2.0-2.1	2.1-2.2	2.2-2.3
**20-24**		230.3 [210.7, 278.3]	282.1 [241.2, 305.2]	318.5 [271.5, 352.1]	326.5 [289.7, 358.9]	355.6 [299.7, 403.6]	383.4 [352.0, 413.5]			
**25-29**			270.0 [228.5, 282.0]	304.5 [273.0, 323.2]	317.7 [273.2, 346.4]	344.3 [313.3, 380.7]	398.6 [370.6, 418.6]	393.9 [329.8, 436.1]		
**30-34**			248.1 [221.3, 279.0]	282.5 [248.2, 308.3]	306.4 [277.4, 341.1]	329.9 [300.7, 375.7]	358.4 [331.9, 400.6]	375.9 [336.8, 397.0]	412.0 [385.1, 437.7]	
**35-39**			261.4 [229.2, 300.0]	287.9 [273.8, 318.8]	297.0 [263.3, 331.4]	334.3 [313.4, 366.7]	365.5 [324.2, 385.1]	342.9 [315.4, 392.9]	409.2 [359.3, 466.9]	
**40-44**		202.4 [139.8, 230.7]	240.4 [184.8, 279.7]	273.3 [239.4, 298.5]	295.5 [265.8, 328.8]	331.7 [297.4, 357.0]	350.5 [315.9, 381.8]	363.8 [334.8, 415.2]	375.0 [353.9, 408.4]	
**45-49**		203.0 [184.6, 245.2]	228.9 [191.8, 251.7]	262.8 [230.2, 290.2]	287.7 [260.4, 320.2]	314.7 [280.9, 345.7]	329.7 [300.5, 368.8]	346.3 [308.0, 384.5]	392.6 [339.7, 425.6]	392.3 [351.9, 434.8]
**50-54**		206.4 [179.7, 227.3]	222.1 [189.0, 246.3]	253.4 [222.5, 279.5]	276.6 [248.2, 304.6]	306.5 [277.2, 333.8]	333.4 [295.1, 367.4]	337.4 [295.3, 376.1]	374.0 [332.9, 408.9]	388.0 [341.9, 414.9]
**55-59**		190.3 [170.5, 212.2]	217.6 [186.9, 243.8]	244.5 [213.4, 269.2]	265.9 [239.7, 295.5]	296.8 [267.0, 326.6]	313.9 [274.3, 346.9]	333.3 [297.7, 360.4]	368.1 [325.2, 391.2]	374.1 [353.7, 428.1]
**60-64**	172.7 [122.9, 198.6]	194.1 [175.1, 209.8]	205.6 [177.8, 233.0]	240.8 [214.3, 269.6]	265.9 [237.8, 293.3]	284.5 [253.6, 317.9]	308.7 [274.4, 336.6]	320.9 [290.6, 352.6]	346.5 [309.3, 379.5]	362.5 [325.6, 386.5]
**65-69**	157.1 [141.6, 185.2]	184.9 [168.0, 206.0]	207.1 [179.2, 232.8]	234.9 [209.1, 261.4]	256.9 [227.1, 285.6]	273.2 [239.1, 302.1]	300.6 [263.6, 328.4]	318.8 [291.3, 346.4]	330.2 [292.2, 373.0]	367.1 [337.3, 372.3]
**70-74**	155.5 [134.6, 182.6]	178.7 [153.8, 201.8]	202.9 [180.3, 224.6]	226.7 [198.1, 251.7]	247.6 [221.8, 273.9]	267.2 [238.6, 296.6]	286.0 [255.1, 316.0]	316.5 [268.6, 344.2]	339.9 [286.5, 359.6]	
**75-79**	153.0 [132.4, 170.1]	181.2 [154.6, 202.5]	200.6 [174.9, 221.3]	219.7 [191.8, 244.1]	240.1 [213.1, 264.4]	261.6 [230.7, 290.6]	281.3 [252.2, 310.1]	285.9 [263.3, 316.6]	298.4 [284.7, 339.2]	
**80-84**	151.6 [132.6, 175.8]	175.2 [155.8, 196.6]	197.5 [175.0, 221.3]	218.6 [193.2, 242.6]	238.0 [210.0, 261.5]	257.4 [229.2, 281.4]	274.3 [248.2, 303.1]	295.2 [255.8, 337.5]		
**85-89**	158.6 [139.9, 181.0]	170.5 [149.0, 192.2]	192.8 [171.0, 211.8]	210.2 [188.8, 235.1]	225.8 [201.5, 253.9]	246.3 [215.8, 277.3]	269.7 [247.2, 286.4]			

Iliopsoas muscle left (Female)

Age (years) \ BSA (m^2)	1.1-1.2	1.2-1.3	1.3-1.4	1.4-1.5	1.5-1.6	1.6-1.7	1.7-1.8	1.8-1.9	1.9-2.0	2.0-2.1
**20-24**		121.3 [106.3, 132.5]	165.3 [145.3, 175.2]	186.0 [166.0, 200.5]	207.7 [186.1, 221.6]	208.5 [196.6, 218.0]	233.7 [209.7, 251.3]			
**25-29**		128.4 [117.7, 147.0]	158.7 [146.7, 167.6]	184.9 [164.4, 197.3]	185.2 [174.3, 213.9]	199.4 [180.6, 218.1]	221.6 [196.3, 233.2]	231.0 [212.4, 252.3]		
**30-34**		120.7 [102.4, 136.4]	159.0 [145.0, 170.6]	173.8 [159.1, 193.5]	188.5 [168.5, 204.3]	204.7 [195.0, 222.2]	217.3 [201.2, 244.5]	227.4 [204.7, 236.7]	279.8 [268.3, 305.7]	
**35-39**		129.8 [107.5, 137.6]	161.9 [141.9, 173.3]	177.6 [163.9, 193.4]	190.8 [171.9, 207.7]	203.7 [191.7, 221.3]	222.5 [204.0, 249.6]	236.9 [217.0, 251.2]	225.6 [182.3, 251.7]	
**40-44**		124.7 [109.0, 146.5]	157.7 [136.8, 180.0]	174.9 [153.9, 191.9]	189.1 [171.6, 203.2]	202.3 [182.7, 222.2]	213.6 [195.4, 233.7]	227.5 [212.4, 253.1]	237.2 [212.6, 268.7]	256.5 [233.3, 290.7]
**45-49**		133.4 [115.0, 147.3]	154.2 [134.1, 169.7]	166.4 [148.7, 185.5]	181.8 [163.9, 197.3]	194.9 [176.7, 213.0]	208.7 [190.8, 228.7]	219.9 [199.5, 240.9]	231.0 [211.0, 266.3]	255.0 [224.5, 277.1]
**50-54**		132.1 [112.1, 139.1]	144.0 [125.6, 168.2]	163.3 [145.5, 178.4]	177.6 [162.4, 194.7]	189.8 [171.6, 207.0]	201.1 [181.3, 218.8]	210.6 [186.2, 235.7]	239.9 [221.3, 262.2]	243.0 [208.3, 254.8]
**55-59**		125.1 [108.9, 143.0]	146.1 [130.8, 162.1]	160.9 [143.2, 177.5]	172.6 [154.8, 189.5]	181.5 [163.9, 200.8]	200.0 [177.0, 221.1]	215.0 [193.4, 238.6]	229.3 [216.5, 238.5]	
**60-64**	113.9 [98.1, 125.1]	130.1 [110.6, 149.8]	143.3 [126.3, 158.4]	156.3 [141.1, 173.9]	168.8 [148.9, 186.6]	181.4 [164.3, 202.8]	192.2 [174.7, 208.8]	209.0 [184.6, 229.2]	214.6 [194.5, 231.3]	228.8 [213.1, 255.5]
**65-69**	116.4 [100.9, 124.0]	122.3 [102.9, 137.9]	142.9 [126.0, 156.1]	152.2 [133.1, 169.7]	165.3 [145.9, 181.9]	177.8 [156.9, 195.6]	192.6 [175.8, 215.0]	207.2 [178.1, 220.0]	216.6 [203.2, 230.1]	
**70-74**	117.9 [99.2, 123.8]	125.2 [107.4, 139.5]	137.6 [118.9, 153.3]	149.6 [132.2, 166.9]	161.1 [144.2, 180.2]	176.9 [155.2, 194.7]	190.5 [164.9, 210.5]	200.0 [170.0, 216.9]	196.6 [182.8, 225.3]	
**75-79**	113.2 [88.2, 129.5]	128.8 [110.2, 143.8]	136.2 [119.4, 150.7]	149.2 [131.8, 165.4]	160.1 [141.3, 178.0]	174.2 [154.2, 193.6]	189.9 [166.0, 206.1]	187.6 [163.5, 205.5]		
**80-84**	112.3 [94.9, 123.6]	126.1 [111.9, 141.8]	135.6 [120.1, 151.7]	150.6 [132.2, 164.3]	160.4 [139.4, 178.3]	172.5 [154.0, 192.5]	178.4 [153.2, 201.0]			
**85-89**	107.1 [85.6, 126.2]	124.7 [108.1, 137.1]	134.8 [116.7, 150.9]	146.3 [128.0, 163.8]	158.5 [136.8, 177.5]	176.3 [151.8, 189.1]	182.4 [172.4, 209.6]			

Iliopsoas muscle total (Male)

Age (years) \ BSA (m^2)	1.3-1.4	1.4-1.5	1.5-1.6	1.6-1.7	1.7-1.8	1.8-1.9	1.9-2.0	2.0-2.1	2.1-2.2	2.2-2.3
**20-24**		456.6 [434.1, 549.9]	546.0 [488.8, 627.7]	618.9 [547.3, 689.2]	639.0 [584.8, 709.0]	706.0 [595.2, 802.0]	741.7 [704.4, 809.8]			
**25-29**			546.4 [448.8, 561.1]	598.4 [547.4, 653.1]	626.2 [550.5, 688.3]	684.7 [623.3, 763.6]	767.3 [719.6, 847.1]	763.1 [634.5, 835.8]		
**30-34**			484.4 [455.1, 573.8]	557.5 [488.6, 605.3]	604.3 [549.9, 672.7]	654.2 [594.5, 733.6]	704.4 [641.1, 794.7]	750.0 [658.1, 778.8]	832.4 [755.5, 864.9]	
**35-39**			517.6 [463.7, 593.2]	563.8 [523.6, 624.1]	589.7 [524.5, 655.8]	662.7 [607.7, 719.1]	719.6 [651.0, 762.7]	679.3 [629.3, 758.5]	789.3 [686.7, 929.0]	
**40-44**		394.8 [278.9, 442.9]	480.2 [370.5, 562.3]	542.1 [476.2, 601.6]	589.6 [539.1, 649.9]	652.3 [584.4, 708.0]	693.0 [625.8, 744.8]	730.3 [650.9, 816.0]	748.2 [693.7, 801.9]	
**45-49**		411.8 [376.9, 481.2]	437.0 [377.4, 511.9]	531.4 [451.8, 571.5]	576.5 [514.7, 638.0]	619.6 [555.0, 682.0]	662.1 [597.3, 726.8]	687.4 [610.2, 749.3]	757.2 [674.4, 829.3]	780.4 [679.8, 833.0]
**50-54**		424.1 [361.7, 460.8]	447.3 [391.0, 487.5]	501.8 [448.1, 548.1]	547.6 [493.0, 601.1]	602.0 [546.8, 655.5]	654.8 [576.7, 719.7]	657.2 [586.4, 738.6]	740.1 [651.3, 804.1]	755.7 [659.8, 836.6]
**55-59**		377.4 [344.1, 422.4]	438.7 [377.3, 482.3]	489.8 [424.5, 532.0]	525.1 [472.1, 578.6]	582.3 [525.2, 642.6]	614.6 [542.0, 675.3]	656.1 [585.9, 715.2]	717.5 [636.9, 761.8]	719.4 [678.5, 809.7]
**60-64**	342.1 [287.0, 399.3]	385.8 [330.4, 416.6]	413.0 [357.3, 461.0]	477.2 [425.6, 527.8]	523.0 [469.2, 576.9]	559.7 [500.9, 621.8]	605.0 [539.2, 657.5]	626.6 [566.5, 691.3]	675.0 [598.7, 749.3]	695.4 [658.8, 755.9]
**65-69**	321.5 [291.4, 379.8]	366.7 [335.8, 416.0]	410.0 [364.5, 459.4]	467.5 [412.5, 516.1]	503.9 [451.1, 558.6]	533.2 [471.3, 587.9]	584.6 [519.7, 643.8]	620.8 [556.8, 666.7]	638.8 [558.6, 726.7]	710.9 [662.7, 777.9]
**70-74**	316.6 [285.7, 357.1]	350.1 [307.3, 395.2]	403.3 [358.7, 444.0]	444.3 [395.0, 492.8]	484.3 [435.0, 535.4]	520.8 [466.5, 575.6]	557.7 [496.5, 613.9]	617.6 [535.8, 673.2]	651.6 [596.0, 692.8]	
**75-79**	308.7 [273.7, 344.0]	357.1 [317.2, 400.7]	398.7 [347.8, 439.5]	430.8 [378.7, 478.6]	470.2 [418.6, 519.1]	508.2 [452.3, 566.7]	545.5 [488.7, 601.3]	555.4 [519.6, 614.6]	603.1 [548.4, 661.1]	
**80-84**	302.9 [274.5, 352.8]	348.9 [312.5, 389.6]	389.5 [346.8, 433.0]	428.9 [381.9, 471.2]	460.8 [412.3, 507.3]	500.4 [448.8, 548.7]	533.1 [486.8, 588.0]	562.8 [460.3, 635.4]		
**85-89**	316.3 [288.3, 360.0]	339.2 [297.6, 381.3]	384.4 [341.0, 421.6]	414.1 [368.3, 460.1]	438.2 [395.2, 487.5]	477.7 [424.0, 532.7]	527.9 [484.6, 567.3]			

Iliopsoas muscle total (Female)

Age (years) \ BSA (m^2)	1.1-1.2	1.2-1.3	1.3-1.4	1.4-1.5	1.5-1.6	1.6-1.7	1.7-1.8	1.8-1.9	1.9-2.0	2.0-2.1
**20-24**		240.2 [220.7, 261.2]	324.4 [289.3, 345.1]	370.2 [332.8, 400.8]	403.5 [364.6, 428.6]	417.9 [380.5, 429.1]	466.1 [407.8, 498.3]			
**25-29**		254.6 [225.3, 292.3]	312.6 [286.3, 327.7]	366.4 [324.2, 392.4]	372.0 [341.7, 423.5]	393.8 [346.5, 426.8]	427.1 [377.0, 463.5]	455.5 [425.5, 499.5]		
**30-34**		229.9 [197.0, 266.1]	317.5 [289.5, 338.6]	347.8 [312.0, 375.7]	372.6 [336.4, 407.6]	414.4 [389.7, 436.6]	416.4 [390.5, 469.0]	446.1 [404.2, 471.1]	565.2 [519.2, 609.5]	
**35-39**		255.7 [214.5, 284.6]	316.3 [287.0, 344.1]	353.9 [321.3, 380.6]	374.8 [338.9, 409.7]	399.6 [374.7, 438.1]	441.2 [396.4, 486.7]	461.8 [417.5, 484.7]	431.5 [361.4, 489.7]	
**40-44**		253.8 [218.6, 289.1]	309.5 [278.8, 355.4]	345.3 [304.3, 379.0]	372.6 [340.2, 400.2]	399.2 [362.1, 432.3]	427.0 [383.9, 452.1]	452.0 [414.1, 502.9]	467.4 [437.0, 517.2]	487.6 [452.6, 556.4]
**45-49**		270.0 [237.6, 296.6]	304.2 [272.2, 333.5]	328.3 [294.9, 366.7]	361.5 [325.6, 389.8]	386.4 [346.3, 414.7]	409.3 [379.5, 445.4]	431.6 [391.5, 476.1]	447.5 [415.7, 511.6]	494.9 [444.0, 525.3]
**50-54**		263.9 [217.2, 277.5]	288.5 [251.5, 326.3]	322.6 [292.4, 354.5]	351.4 [318.5, 383.2]	374.3 [336.7, 407.6]	391.9 [355.9, 433.1]	415.8 [364.6, 465.6]	462.4 [428.3, 507.5]	469.5 [416.2, 481.5]
**55-59**		253.2 [217.0, 284.1]	291.7 [262.1, 320.0]	317.5 [284.4, 348.2]	339.8 [305.9, 371.5]	357.8 [322.9, 393.2]	391.1 [349.1, 427.9]	423.6 [381.1, 466.6]	440.0 [406.7, 473.7]	
**60-64**	229.7 [212.6, 260.1]	253.3 [225.9, 295.0]	285.1 [251.3, 310.7]	310.4 [282.5, 342.1]	331.7 [294.6, 367.6]	354.1 [321.8, 398.7]	371.2 [347.4, 406.0]	406.3 [369.7, 443.8]	423.2 [384.0, 455.7]	457.9 [389.7, 491.2]
**65-69**	228.4 [194.2, 258.8]	240.3 [220.3, 274.2]	280.9 [251.5, 309.5]	301.9 [263.0, 333.6]	324.3 [290.8, 355.7]	348.3 [308.6, 384.2]	378.1 [340.3, 414.2]	396.9 [357.9, 423.3]	417.2 [390.2, 442.6]	
**70-74**	222.3 [200.8, 250.7]	248.5 [214.1, 272.2]	273.4 [239.1, 302.5]	296.4 [262.1, 327.7]	317.5 [286.7, 352.0]	342.1 [303.1, 378.6]	374.4 [324.3, 402.0]	384.5 [337.5, 420.3]	376.6 [353.8, 443.8]	
**75-79**	228.0 [187.4, 263.8]	253.5 [220.2, 283.7]	267.2 [235.0, 295.4]	290.3 [259.8, 321.9]	315.5 [276.8, 346.2]	340.9 [304.7, 376.7]	365.7 [331.0, 402.6]	357.6 [326.1, 382.1]		
**80-84**	224.1 [190.0, 259.9]	250.0 [225.0, 281.2]	265.7 [236.4, 295.5]	292.3 [261.5, 321.3]	313.9 [276.5, 346.1]	333.0 [299.1, 374.9]	339.6 [304.0, 391.9]			
**85-89**	209.8 [178.1, 254.3]	247.2 [220.3, 270.2]	266.4 [229.6, 294.6]	286.6 [251.3, 317.7]	304.7 [272.1, 341.1]	340.6 [294.3, 364.3]	347.9 [341.5, 392.7]			
